# Dependency of Heterochromatin Domains on Replication Factors

**DOI:** 10.1534/g3.117.300341

**Published:** 2017-11-29

**Authors:** Leonie Johanna Jahn, Bethany Mason, Peter Brøgger, Tea Toteva, Dennis Kim Nielsen, Genevieve Thon

**Affiliations:** Department of Biology, University of Copenhagen, BioCenter, 2200, Denmark

**Keywords:** chromatin remodeling, cohesins, DNA replication, fission yeast, gene silencing, genetic screen, heterochromatin, mating-type region, nuclear organization, proline isomerase

## Abstract

Chromatin structure regulates both genome expression and dynamics in eukaryotes, where large heterochromatic regions are epigenetically silenced through the methylation of histone H3K9, histone deacetylation, and the assembly of repressive complexes. Previous genetic screens with the fission yeast *Schizosaccharomyces pombe* have led to the identification of key enzymatic activities and structural constituents of heterochromatin. We report here on additional factors discovered by screening a library of deletion mutants for silencing defects at the edge of a heterochromatic domain bound by its natural boundary—the *IR-R*^+^ element—or by ectopic boundaries. We found that several components of the DNA replication progression complex (RPC), including Mrc1/Claspin, Mcl1/Ctf4, Swi1/Timeless, Swi3/Tipin, and the FACT subunit Pob3, are essential for robust heterochromatic silencing, as are the ubiquitin ligase components Pof3 and Def1, which have been implicated in the removal of stalled DNA and RNA polymerases from chromatin. Moreover, the search identified the cohesin release factor Wpl1 and the forkhead protein Fkh2, both likely to function through genome organization, the Ssz1 chaperone, the Fkbp39 proline *cis-trans* isomerase, which acts on histone H3P30 and P38 in *Saccharomyces cerevisiae*, and the chromatin remodeler Fft3. In addition to their effects in the mating-type region, to varying extents, these factors take part in heterochromatic silencing in pericentromeric regions and telomeres, revealing for many a general effect in heterochromatin. This list of factors provides precious new clues with which to study the spatiotemporal organization and dynamics of heterochromatic regions in connection with DNA replication.

The various chromatin states found in eukaryotes fall into two histologically distinct types: euchromatin, which sustains gene expression, and heterochromatin, with repressive functions. While numerous forms of euchromatin can be distinguished based on histone modifications, heterochromatin is more homogenously characterized by the methylation of histone H3 at lysine 9 (H3K9me). H3K9me silences repeats and transposable elements that together constitute more than half of the human genome (Gregory 2005), and recent investigations of embryonic stem cells and cell reprogramming have further shown the importance of H3K9me in maintaining cell identity in differentiated cells through the formation of facultative heterochromatin (Becker *et al.* 2016). Beyond the control of gene expression, chromatin fulfills structural roles and participates in DNA recombination, and in the repair, replication, and condensation of chromosomes. How these various functions are coordinated to accommodate each other at the chromatin level is an important aspect of eukaryotic genome biology.

Much knowledge of chromatin structure and function stems from studies of the fission yeast *Schizosaccharomyces pombe*. Genetic screens in this organism produced mutants of the H3K9 methyltransferase Clr4 (Ekwall and Ruusala 1994; Thon *et al.* 1994), which together with its metazoan homolog SUVAR39 was the first identified histone methyltransferase (Rea *et al.* 2000). Clr4 and SUVAR39 establish large heterochromatic domains. The genetic screens that identified the *clr4* locus relied on derepression of the silent mating-type loci and nearby reporters. Not only do *clr4* mutants express these normally silent genes due to loss of H3K9me heterochromatin, but several aspects of mitotic and meiotic recombination are also altered in the mutants (Thon *et al.* 1994; Ellermeier *et al.* 2010). Thus, the system provides an example where chromatin structure coordinately controls gene expression and recombination through H3K9me. In addition to Clr4, genetic screens have produced mutants in: the HP1-like protein Swi6 (Gutz and Schmidt 1985; Lorentz *et al.* 1994); the Clr7 (a.k.a. Raf2, Cmc2, and Dos2), Clr8 (a.k.a. Raf1, Cmc1, and Dos1), and Rik1 subunits of the Clr4-associated complex CLRC (Li *et al.* 2005; Thon *et al.* 2005); the Clr1, Clr2, and Clr3 subunits of the SHREC histone deacetylase complex (Thon and Klar 1992; Ekwall and Ruusala 1994; Thon *et al.* 1994; Sugiyama *et al.* 2007; Job *et al.* 2016); Clr5 (Hansen *et al.* 2011); the Clr6 histone deacetylase (Grewal *et al.* 1998); and the Ubc4 E2 ubiquitin ligase (Irvine *et al.* 2009). This mutant assortment mirrors mutants isolated in *Drosophila* (Elgin and Reuter 2013) and mouse (Blewitt *et al.* 2005), and emphasizes the evolutionarily conserved properties of heterochromatin, such as H3K9me and histone hypoacetylation.

Major players in *S. pombe* heterochromatin formation were identified by the screens mentioned above, although not all players. This is partly because some requirements for heterochromatin formation are location-specific. At centromeres, heterochromatin is strongly driven by RNA interference (RNAi), whereas this is less the case in the mating-type region (Ekwall *et al.* 1999; Volpe *et al.* 2002). The formation of telomeric heterochromatin depends on RNAi and on the Shelterin protein Taz1 (Kanoh *et al.* 2005; Hansen *et al.* 2006). Small heterochromatic islands dispersed in the genome depend on Red1 or Shelterin components (Hiriart *et al.* 2012; Zofall *et al.* 2016). In addition, some factors possibly eluded original screens due to their relatively small contribution to heterochromatic gene silencing, this would be the case for the Swi6 paralog Chp2 (Thon and Verhein-Hansen 2000), the impaired growth of the mutants, as for the Pcu4 cullin (Thon *et al.* 2005), or to functional redundancy, as for Atf1/Pcr1 redundant with RNAi to establish heterochromatin in the mating-type region (Jia *et al.* 2004). Also, the screens were not saturated, as some loci appeared as mutational hotspots while others were underrepresented. Other loci affecting heterochromatin formation or function were subsequently identified in candidate approaches (references in Table 1). Recently, a screen of the Bioneer deletion library recapitulated some of these findings for pericentromeric silencing by isolating factors required for the silencing of an *ade6^+^* reporter placed in a *dg* repeat of centromere 1 (Bayne *et al.* 2014).

**Table 1:** List of silencing factors identified in this study, with references to prior identification in genetic screens or candidate and biochemical approaches

Gene ID	Product	Description	Reference
Histone deacetylase complexes			
SPBC2D10.17	Clr1	SHREC complex cryptic loci regulator 1 subunit	Thon and Klar (1992), Sugiyama *et al.* (2007)
SPAC1B3.17	Clr2	SHREC complex cryptic loci regulator 2 subunit	Ekwall and Ruusala (1994), Thon *et al.* (1994), Sugiyama *et al.* (2007)
SPBC800.03	Clr3	SHREC complex cryptic loci regulator 3 subunit (histone deacetylase)	Ekwall and Ruusala (1994), Thon *et al.* (1994), Sugiyama *et al.* (2007)
SPBP35G2.10	Mit1	SHREC complex ATP-dependent DNA helicase subunit Mit1	Sugiyama *et al.* (2007)
SPAC16C9.05	Cph1	Clr6 histone deacetylase-associated PHD protein-1	Nicolas *et al.* (2007)
Histone H3K9 methyltransferase complex CLRC and Dos2 complexes			
SPBC428.08c	Clr4	Cryptic loci regulator 4 (histone H3K9 methyltransferase)	Ekwall and Ruusala (1994), Thon *et al.* (1994), Hong *et al.* (2005), Horn *et al.* (2005)
SPCC970.07c	Raf2/Dos2/Cmc2/Clr7	Rik1-associated factor 2/delocalization of Swi6 factor 2/cullin-associated methyltransferase complex protein 2/cryptic loci regulator 7	Hong *et al.* (2005), Horn *et al.* (2005), Li *et al.* (2005), Thon *et al.* (2005)
SPCC613.12c	Raf1/Dos1/Cmc1/Clr8	Rik1-associated factor 1/delocalization of Swi6 factor 2/cullin-associated methyltransferase complex protein 1/cryptic loci regulator 8	Hong *et al.* (2005), Horn *et al.* 2005), Li *et al.* (2005), Thon *et al.* (2005)
SPCC11E10.08	Rik1	Silencing protein Rik1	Ekwall and Ruusala (1994), Hong *et al.* (2005), Horn *et al.* (2005), Thon *et al.* (2005)
SPAC1071.02	Mms19	Dos2 silencing complex subunit	Li *et al.* (2011)
HP1 family and associated factors			
SPAC664.01c	Swi6	Chromodomain protein Swi6	Gutz and Schmidt (1985)
SPAC1851.03	Ckb1	CK2 family regulatory subunit	Shimada *et al.* (2009), Braun *et al.* (2011)
CRL4			
SPAC17H9.19c	Cdt2	WD repeat protein Cdt2	Braun *et al.* (2011), Bayne *et al.* (2014)
SPAC17H9.10c	Ddb1	Damaged DNA binding protein 1	Braun *et al.* (2011), Bayne *et al.* (2014)
CSN			
SPBC215.03c	Csn1	COP9 signalosome complex subunit Csn1	Bayne *et al.* (2014)
RPC			
SPAC694.06c	Mrc1	Mediator of replication checkpoint 1	
SPAPB1E7.02c	Mcl1	DNA polymerase α accessory factor	Mamnun *et al.* (2006), Natsume *et al.* (2008)
SPBC216.06c	Swi1	Replication fork protection complex subunit Swi1	
SPBC30D10.04	Swi3	Replication fork protection complex subunit Swi3	
SPBC609.05	Pob3	FACT complex subunit	Lejeune *et al.* (2007)
Ubiquitin–SUMO-related			
SPCC338.16	Pof3	F-box protein	Katayama *et al.* (2002), Mamnun *et al.* (2006)
SPBC354.10	Def1	RNAPII degradation factor (predicted)	
SPAC1687.05	Pli1	SUMO E3 ligase	Xhemalce *et al.* (2004)
Genome organization/replication timing			
SPBC16G5.15c	Fkh2	Forkhead transcription factor Fkh2	
SPBC428.17c	Wpl1	Cohesin loading/unloading factor (WAPL) Wpl1	
SPAC6F6.17	Rif1	Telomere length regulator protein Rif1	Toteva *et al.* (2017)
SPBC1198.^11^C	Reb1	RNA polymerase I transcription termination factor /replication block	Jakociunas *et al.* (2013)
Chromatin remodelers/chaperones			
SPAC25A8.01c	Fft3	Fission yeast fun 30 related protein Fft3 ATP-dependent helicase, chromatin remodeler	Stralfors *et al.* (2011)
SPAC31G5.19	Abo1	ATPase with bromodomain protein	Gal *et al.* (2016)
SPBC1347.02	Fkpb39	FKBP-type peptidyl-prolyl *cis*-trans isomerase (predicted)	
SPAC57A7.12	Ssz1	Heat shock protein	
Histone acetyltransferase			
SPAC1952.05	Gcn5	SAGA complex histone acetyltransferase catalytic subunit Gcn5	
RNA-associated proteins			
SPAC4F10.14c	Btf3	Nascent polypeptide-associated complex β subunit/basic transcription factor Btf3	
SPCC4G3.15c	Not2	CCR4-NOT complex NOT box subunit Not2	Cotobal *et al.* (2015)
SPAC29B12.06c	Rcd1	CCR4-NOT complex RNA-binding protein subunit Rcd1	Cotobal *et al.* (2015)
Miscellaneous			
SPBC428.07	Meu6	Meiotic chromosome segregation protein Meu6 (adjacent to *clr4*^+^ gene)	

ID, identifier; PHD, Plant homeodomain; CLRC, Clr4 complex; FACT, facilitates chromatin transcription; RNAPII, RNA polymerase II.

Comprehensive models for heterochromatin formation and maintenance have been derived from the phenotypes of *S. pombe* mutants, with noncoding RNAs, RNAi, and DNA-binding proteins participating in the establishment of heterochromatic domains whose subsequent maintenance invokes epigenetic components. Domains in the 20–100 kb range are formed at centromeres, telomeres, and the mating-type region. Smaller heterochromatin patches are formed at internal chromosomal locations, some of which participate in the repression of meiotic genes during vegetative growth.

The new screens reported here build on our recent identification of DNA elements capable of functionally replacing the natural chromatin boundary, *IR-R^+^*, at the edge of the mating-type region (Jakociunas *et al.* 2013; Toteva *et al.* 2017). *IR-R^+^* deletion leads to reduced heterochromatic silencing (Thon *et al.* 2002) that can be restored by artificial boundaries. Thus, five genomic elements, whose boundary activity requires the regulators of replication origin firing Taz1 and Rif1, can substitute for *IR-R^+^* (*STAR1-5*, for Sensitive to Taz1 and Rif1), as well as another uncharacterized element originating from a unique chromosomal location, *BTH1* (Boundary To Heterochromatin 1) (Toteva *et al.* 2017). At their endogenous chromosomal locations, *STAR* elements are adjacent to late replication origins. They are physically bound by Taz1, which, together with Rif1, delays origin firing (Hayano *et al.* 2012; Tazumi *et al.* 2012). The effects of Taz1, Rif1, and *STAR* elements on replication and chromatin structure appear to be mechanistically connected, perhaps through protein phosphatase 1 (Toteva *et al.* 2017). rDNA repeats can also form ectopic boundaries, in this case highlighting the essential role of the replication-blocking protein Reb1 in boundary formation (Jakociunas *et al.* 2013). Additional phenotypes suggest coordinated control of DNA replication and boundary positioning. In particular, the heterochromatic part of the mating-type region replicates early in S phase in contrast to the flanking euchromatin. Deletion of the *IR-R*^+^ boundary perturbs the replication pattern at the edge of the domain in addition to perturbing heterochromatin (Toteva *et al.* 2017). Our new screens confirm and extend these previous observations by showing that multiple proteins associated with the replisome participate in heterochromatic gene silencing. This is in addition to several other factors with hitherto undocumented roles in heterochromatin, whose identification further increases our understanding of the interplay between genome organization and gene expression.

## Materials and Methods

### Strains, genetic manipulations, media, and oligonucleotides

The *S. pombe* strains used in this study are listed in Supplemental Material, Table S3 in File S1. Oligonucleotide sequences are provided in Table S4 in File S1. Media and yeast handling were conducted according to Ekwall and Thon (2017). NBA medium (Yeast Nitrogen Base) is identical to *Saccharomyces cerevisiae* SC medium.

### Screens of Bioneer deletion library

An *S. pombe* deletion library of haploid strains (Bioneer, version 3) was screened in an arrayed format with the help of a ROTOR HDA robot (Singer Instruments), as described in Toteva *et al.* (2017) and based on Baryshnikova *et al.* (2010). Compared to Toteva *et al.* (2017), where the B6 and B7 strains were used as query strains, the following additional strains were mated here to the library: B14, B15, B16, B17, B18, and B19. In all cases, recombinant progeny of the crosses were selected for G418 resistance, conferred by the ORF deletions, and uracil prototrophy, conferred by the mating-type region of the query strains. *(EcoRV)*::*ade6*^+^ derepression was tested by growth in the absence of adenine, first in two rounds of high-throughput screening and subsequently by spot tests of a ten-fold dilution series for potential hits. Isolated colonies for the best-looking hits were obtained by streaking Bioneer library strains. The purified strains were then checked by PCR to confirm the expected ORF deletions, using Taq polymerase and primer Cp-N10 or Cp-C3, which anneal within the *kanR* cassette (Kim *et al.* 2010), in combination with loci-specific primers. Cp-N10 was used with Cp5 loci-specific primers and Cp-C3 with Cp3 loci-specific primers. Once confirmed, the purified strains were crossed again with the query strains to test the resulting Ade phenotype in *ade6-DN/N* progeny. *ade6-DN/N* was identified by PCR on genomic DNA with primers GTO-232 and GTO-233.

### Construction of mcl1, wpl1, and dpb4 deletion strains

To retest the phenotypes of *mcl1*Δ::*kanR (SPAPB1E7.02c*Δ), *wpl1*Δ::*kanR* (*SPBC428.17c*Δ), and *dpb4*Δ::*kanR* (*SPBC3D6.09*Δ), deletion cassettes were amplified from the corresponding Bioneer deletion library strains, respectively: V3-P19-76, V3-^32^P-77, and V3-P02-69, with the oligonucleotide pairs GTO-1332 and GTO-1333, GTO-1365 and GTO-1366, and GTO-1363 and GTO-1364, and Phusion HF DNA polymerase (Thermo Fisher Scientific). The *mcl1*Δ::*kanR* amplicon was cloned into the *Eco*RV site of plasmid pJET1.2 (Thermo Fisher Scientific), generating plasmid pPA7. This plasmid was digested with *Bgl*II to release the *mcl1*Δ::*kanR* insert for *S. pombe* transformation, while the purified amplicons for *wpl1*Δ::*kanR* and *dpb4*Δ::*kanR* were used directly. Each deletion was introduced into the *S. pombe* strains PG3950, PC152, PT600, and PG3947, and transformants were selected on YES plates with G418. Gene replacements were confirmed by PCR with primers Cp-N10 or Cp-C3 (Kim *et al.* 2010) employed as above with locus-specific primers: Cp5-mcl1D, Cp5-wpl1D, and Cp5-dpb4D were used with Cp-N10, and Cp3-mcl1D, Cp3-wpl1D, and Cp3-dpb4D were used with Cp-C3. The PG3950, PC152, PT600, and PG3947 transformants with *mcl1*Δ::*kanR* were named PA71, PA72, PA73, and PA74; those with *dpb4*Δ::*kanR* were named PA77, PA78, PA79, PA80, and PA81; and those with *wpl1*Δ::*kanR* were named PA82, PA83, PA84, and PA85. During the construction of these strains, transformants were replica-plated onto medium with limited adenine (YE) and medium lacking adenine (AA-ade) at several steps, including following the original G418 selection of transformants, during the colony purification of multiple candidates to gauge effects on *(EcoRV)*::*ade6*^+^ expression, and to ensure that the phenotypes of the strains saved in the collection were representative.

### RNA preparation and RT-QPCR

*(EcoRV)*::*ade6*^+^ RNA levels were monitored as described previously (Hansen *et al.* 2005; Jakociunas *et al.* 2013). Briefly, for each strain to be examined, three isolated colonies were first patched on YES and subsequently propagated overnight in 50 ml EMM2 cultures supplemented with adenine, leucine, and uracil, to an OD_600_ of 0.4. Cell pellets were flash frozen in liquid nitrogen and stored at −80°. Following RNA extraction, 2 µg RNA were treated with RQ1 DNase (Promega), diluted with DEPC-treated H_2_O to a final volume of 200 µl, and stored at −20°. QPCRs were set up with 7.5 µl DNase-treated RNA samples in 25 µl reactions using a QuantiFast Multiplex RT-PCR kit (QIAGEN) and two primers at 1 µM each. Three technical replicates were set up for each reaction. The reactions were performed as suggested by the manufacturer: 10 min at 50°, followed by 5 min at 95° and 39 cycles of 10 sec at 95°, and 30 sec at 60°, with data capture at each cycle, in a Bio-Rad CFX96 Thermal Cycler. Melting curves were produced on the final products and no RT controls were included. Reactions were set up with the primer pairs GTO-218 and GTO-219 (*ade6*^+^), and TJO-55 and TJO-58 (*act1*^+^). Data were analyzed using Bio-Rad CFX Manager and Microsoft Excel, normalizing *ade6*^+^ expression to *act1*^+^ in all samples. Mean normalized expression (MNE) values were calculated according to the following equation: *MNE* = (*E_act1_*)^CT^*^act1^*^,^ *^mean^*/(*E_ade6_*) ^CT^*^ade6^*^,^ *^mean^*, where *E_act1_* is the efficiency of *act1*^+^ DNA amplification determined on 10-fold serial dilutions of genomic DNA, *CT_act1_*_,_*_mean_* is the mean cycle threshold for *act1*^+^ RNA amplification, *E_ade6_* is the efficiency of amplification of the target of interest (*ade6*^+^) determined on 10-fold serial dilutions of genomic DNA, and *CT_ade6_*_,_*_mean_* is the mean cycle threshold for *ade6*^+^ RNA amplification.

### Data availability

Strains are available upon request. All Supplemental Tables and Figures are regrouped in File S1. Table S1 in File S1 presents a list of Bioneer deletion strains selected for the second and third mutant screens and Table S2 in File S1 gives a list of potential false positives. Figures S1–S8 in File S1 show the phenotypes of mutants examined in the second screen, while Figures S9–S11 in File S1 show the phenotypes of mutants examined in the third screen. Table S3 in File S1 contains a list of strains and their genotypes, while Table S4 in File S1 contains oligonucleotide sequences.

## Results and Discussion

### Screen for factors required for heterochromatin integrity close to domain boundaries

**Figure 1:**
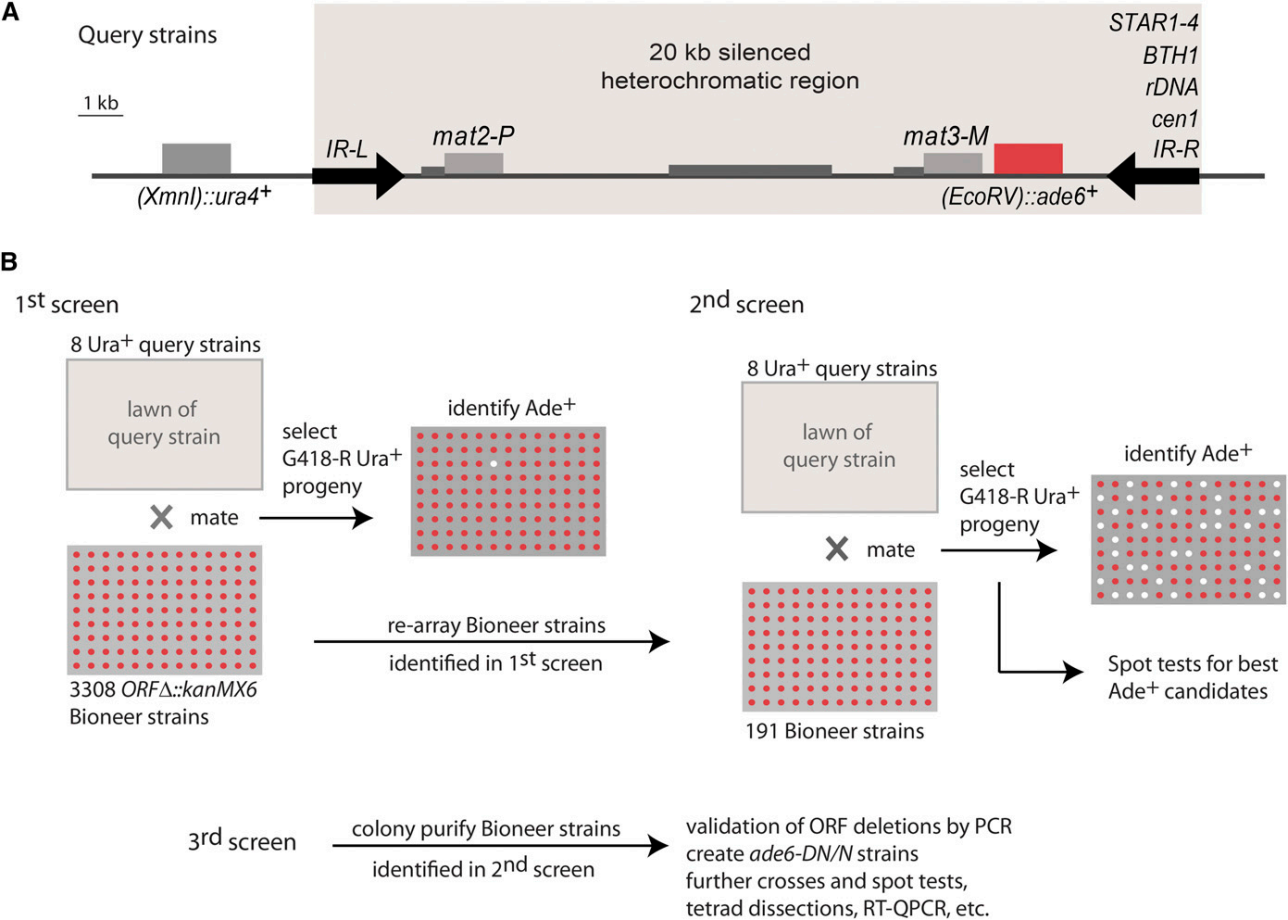
Experimental design. (A) Mating-type region of query strains, showing the *(EcoRV)*::*ade6*^+^ reporter used to monitor heterochromatic silencing close to the wild-type boundary element *IR-R*^+^ or ectopic boundaries. Silencing of *(EcoRV)*::*ade6*^+^ results in red colony formation on the medium used, while expression results in white colonies. The euchromatic *(XmnI):ura4*^+^ gene was used to select the mating-type region in crosses with the Bioneer collection. (B) ORF deletions allowing *(EcoRV)*::*ade6*^+^ expression were identified in three rounds of screening as depicted. Candidates were retested as described in the text. ORF, open reading frame; RT-QPCR, reverse transcription-quantitative polymerase chain reaction.

An arrayed library of 3308 *S. pombe* ORF deletion strains (Kim *et al.* 2010) was used to determine the requirements for heterochromatic gene silencing at the edge of the mating-type region in eight strains: a strain with the wild-type *IR-R^+^* boundary and strains with, respectively: *STAR1-4*, *BTH1*, an *rDNA* repeat, or the edge of *cen1* in the place of *IR-R^+^* (Figure 1A). The eight query strains contained the *(EcoRV)*::*ade6*^+^ reporter gene close to the edge of the heterochromatic domain to provide a readout for gene silencing (Thon *et al.* 1999), and a euchromatic *ura4*^+^ gene tightly linked to the mating-type region to allow for its selection (*(XmnI)*::*ura4*^+^). The query strains were crossed with the deletion library in parallel large-scale experiments. Progeny combining the mating-type region of interest with each ORF deletion were selected according to the scheme in Figure 1B and tested for *(EcoRV)*::*ade6*^+^ expression. Candidate factors whose deletions appeared to permit the expression of *(EcoRV)*::*ade6*^+^ were identified. At this stage, even deletions with a dubious phenotype were chosen for further examination alongside deletions with a potentially strong effect. The corresponding 196 strains from the Bioneer library were rearrayed on a single plate (191 strains) or processed individually (five strains) and retested by genetic crosses against all query strains, leading to a shorter list of 48 factors potentially required for *(EcoRV)*::*ade6*^+^ repression (Figures S1–S8 and Table S1 in File S1). Factors with extensively documented effects on heterochromatin formation were not tested further, and we focused instead on factors whose effects on heterochromatin had not been documented before, or not tested in the mating-type region. Recombinant Ura^+^ G418-resistant progeny were colony-purified and spotted on indicator and selective plates to better evaluate *(EcoRV)*::*ade6*^+^ expression, leading to a short list of 33 candidate genes with a heterochromatic silencing defect in at least one of the queried mating-type regions. For these candidates, single colonies were isolated from the Bioneer collection, the ORF deletions were verified by PCR using locus-specific primers, and we obtained recombinant progeny combining the mating-type regions of interest with each ORF deletion in an *ade6-DN/N* background. The *ade6-DN/N* background is well suited to the monitoring of *ade6*^+^ expression by colony color on a limiting concentration of adenine or by RT-QPCR with primers that specifically recognize *ade6*^+^. In addition to Bioneer strains, three deletion strains that were not present in the library were added to the analysis (*swi1*Δ::*kanR*, *swi3*Δ::*kanR*, and *clr8*Δ::*kanR*).

**Figure 2:**
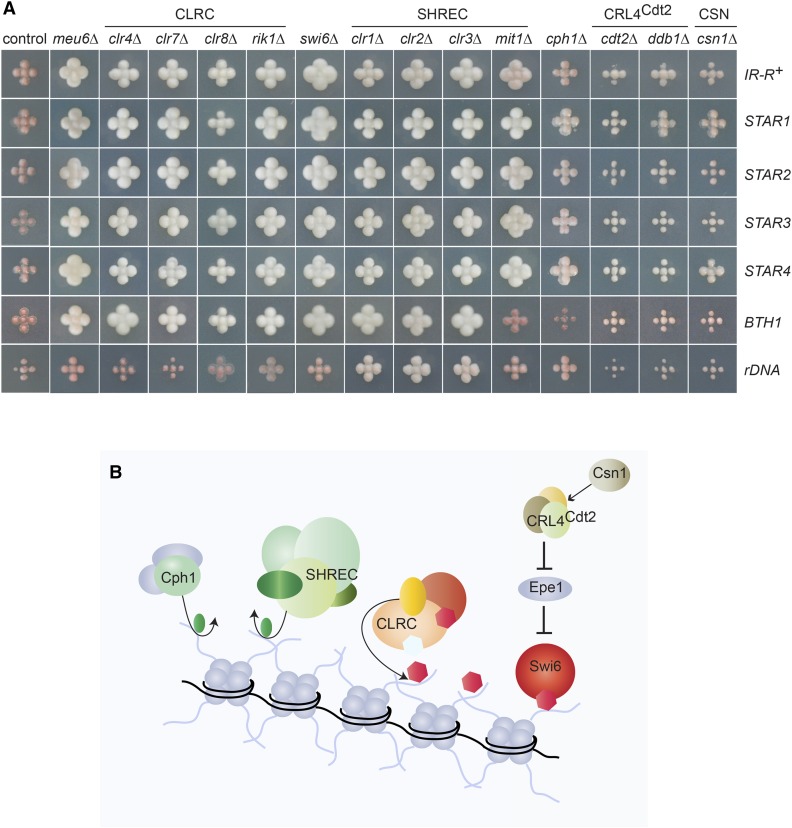
Factors identified in the screens include histone modifiers and readers, and their regulators. (A) Bulk progeny of crosses combining the indicated boundary elements and ORF deletions, replicated onto medium lacking adenine [NBA (yeast nitrogen base) supplemented with leucine] to assay *(EcoRV)*::*ade6*^+^ expression. Light colors reflect *(EcoRV)*::*ade6*^+^ expression. Control: an ORF deletion with no effect on *(EcoRV)*::*ade6*^+^ expression (V3-P25-45, SPAC15E1.07c). (B) Schematic representation of factors and complexes shown in (A). HDACs remove acetyl groups (in green); CLRC methylates H3K9 (in red); CRL4^Cdt2^, positively regulated by Csn1, destabilizes the antisilencing factor Epe1. HDACs, histone deacetylases; ORF, open reading frame.

**Figure 3:**
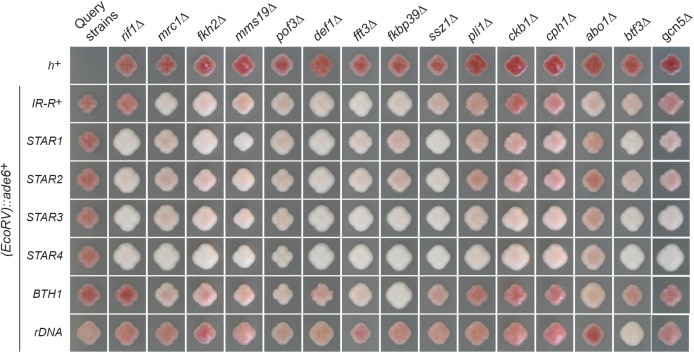
Overview of other factors identified in the screen. Purified *ade6-DN/N* strains combining the indicated boundary elements and open reading frame deletions were propagated on medium lacking adenine [NBA (yeast nitrogen base) supplemented with leucine] to assay *(EcoRV)*::*ade6*^+^ expression. Spot tests for these strains are presented in Figures S9–S11 in File S1.

**Figure 4:**
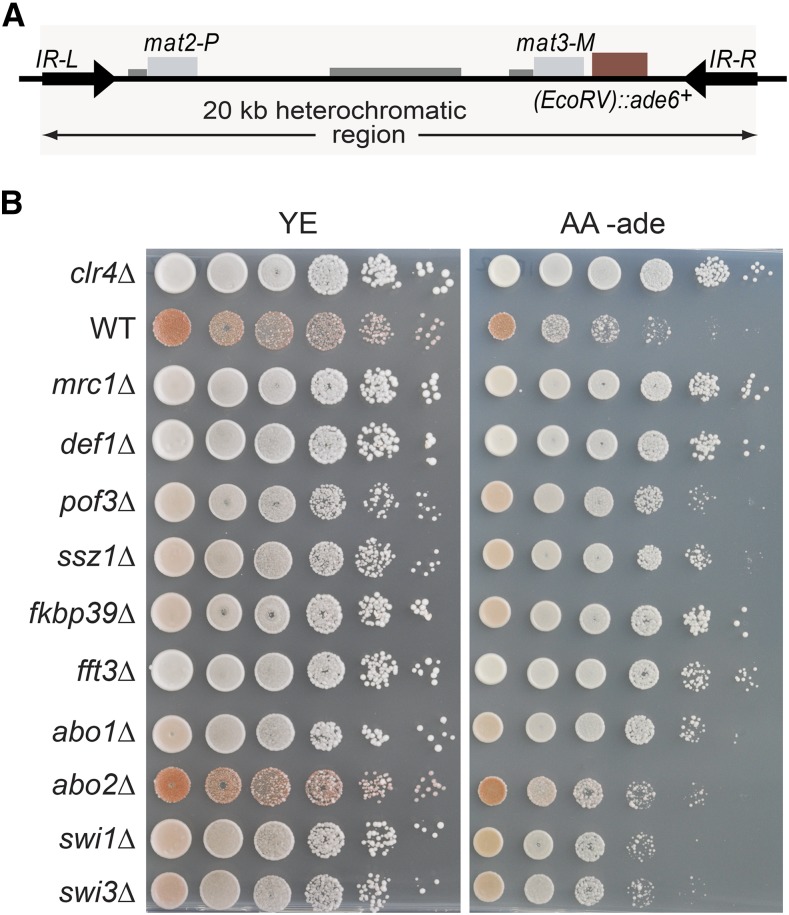
Effects of selected factors on *(EcoRV)*::*ade6*^+^ in the presence of the WT boundary *IR-R*^+^. (A) Mating-type region in this experiment. (B) Growth assays with 10-fold serial dilutions of indicated deletion strains. Strains LJ99, PG3950, LJ102, LJ100, LJ220, LJ203, LJ104, LJ103, LJ199, LJ105, PM20, and PM24 were used. AA -ade, medium lacking adenine; WT, wild-type; YE, medium with limited adenine.

**Figure 5:**
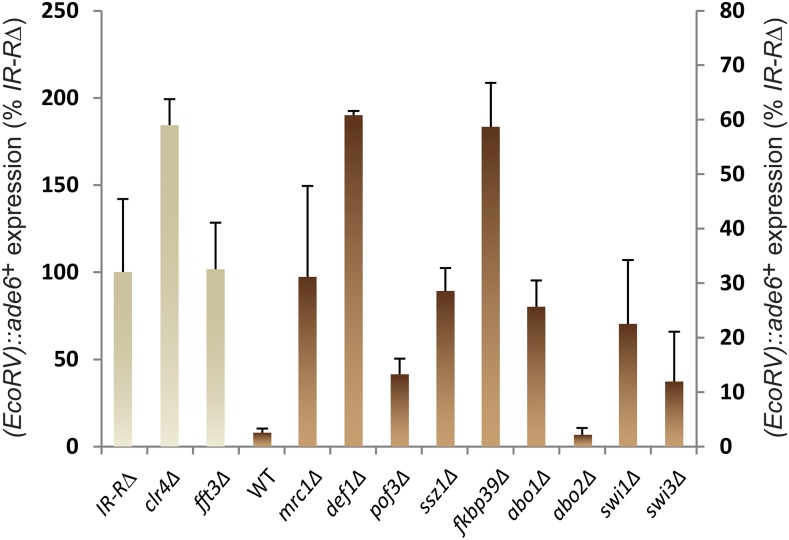
Effects of selected factors on *(EcoRV)*::*ade6*^+^ transcript in the presence of the WT boundary *IR-R*^+^, assayed by RT-QPCR. The strains are the same as in Figure 4. The means of three biological replicates are displayed. *(EcoRV)*::*ade6*^+^ RNA level was normalized to *act1*^+^ in each strain and is expressed as % expression in *IR-R*Δ cells. The *y*-axis labeling on the left applies to the three strains on that side that have the highest *(EcoRV)*::*ade6*^+^ expression. The labeling on the right applies to the other strains. Errors bars represent the SE of the mean. RT-QPCR, reverse transcription-quantitative polymerase chain reaction; WT, wild-type.

**Figure 6:**
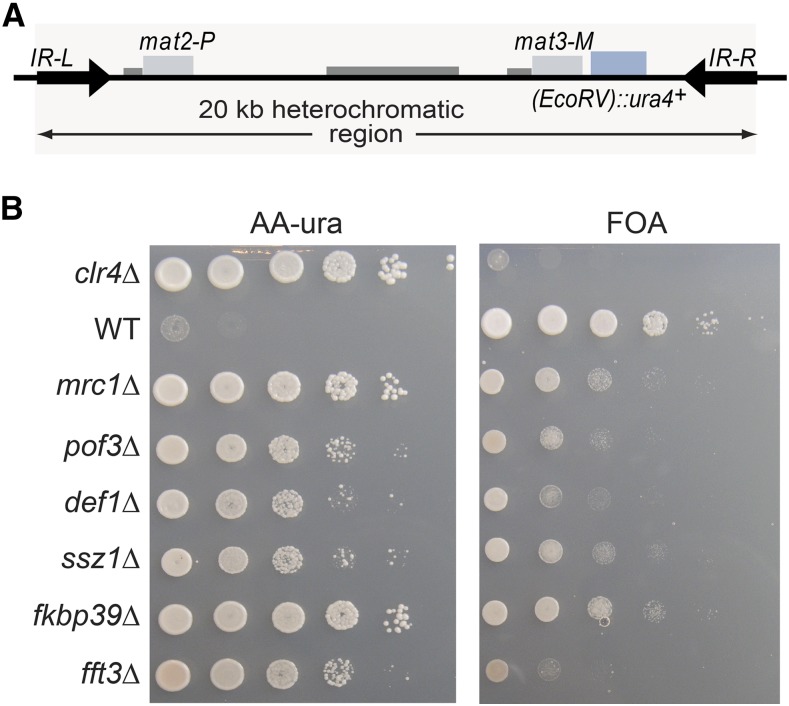
Effects of selected factors on *(EcoRV)*::*ura4*^+^ in the presence of the WT boundary *IR-R*^+^. (A) Mating-type region in this experiment. (B) Growth assays with 10-fold serial dilutions of indicated deletion strains. Strains used for spot tests: LJ177, PG1899, LJ176, LJ178, LJ179, and LJ175. AA-ura, medium lacking uracil; FOA, fluoroorotic acid; WT, wild-type.

Comprehensive spot-test analyses assessing silencing by factors that made it through the entire screen are shown in Figures S9–S11 in File S1. A subset of factors are presented in Figure 2 and Figure 3 for their effects on all query strains, and in Figure 4 and Figure 5 for their effects with the wild-type boundary *IR-R*^+^. Derepression of *(EcoRV)*::*ade6*^+^ occurs in *IR-R*Δ and *clr4*Δ cells, resulting in white or light pink colonies on medium with limiting adenine and improved growth in the absence of adenine. While not as pronounced as for *clr4*Δ, the phenotypes of the other mutations tested were distinctive and were confirmed by quantification of the *(EcoRV)*::*ade6*^+^ transcript (Figure 5). Increases ranging from 5- to 40-fold over wild-type were measured and normalized to *(EcoRV)*::*ade6*^+^ expression in the *IR-R*Δ strain in Figure 5 (*abo2*Δ is shown for comparison with the deletion of its paralog *abo1*Δ). A different reporter, *(EcoRV)*::*ura4*^+^, was affected in the same way. In particular, *mrc1*Δ, *pof3*Δ, *def1*Δ, *ssz1*Δ, *fkbp39*Δ, and *fft3*Δ cells grew better in the absence of uracil than in the presence of fluoroorotic acid (FOA), unlike the parental wild-type strain for which *(EcoRV)*::*ura4*^+^ repression leads to the opposite growth pattern (Figure 6).

**Figure 7:**
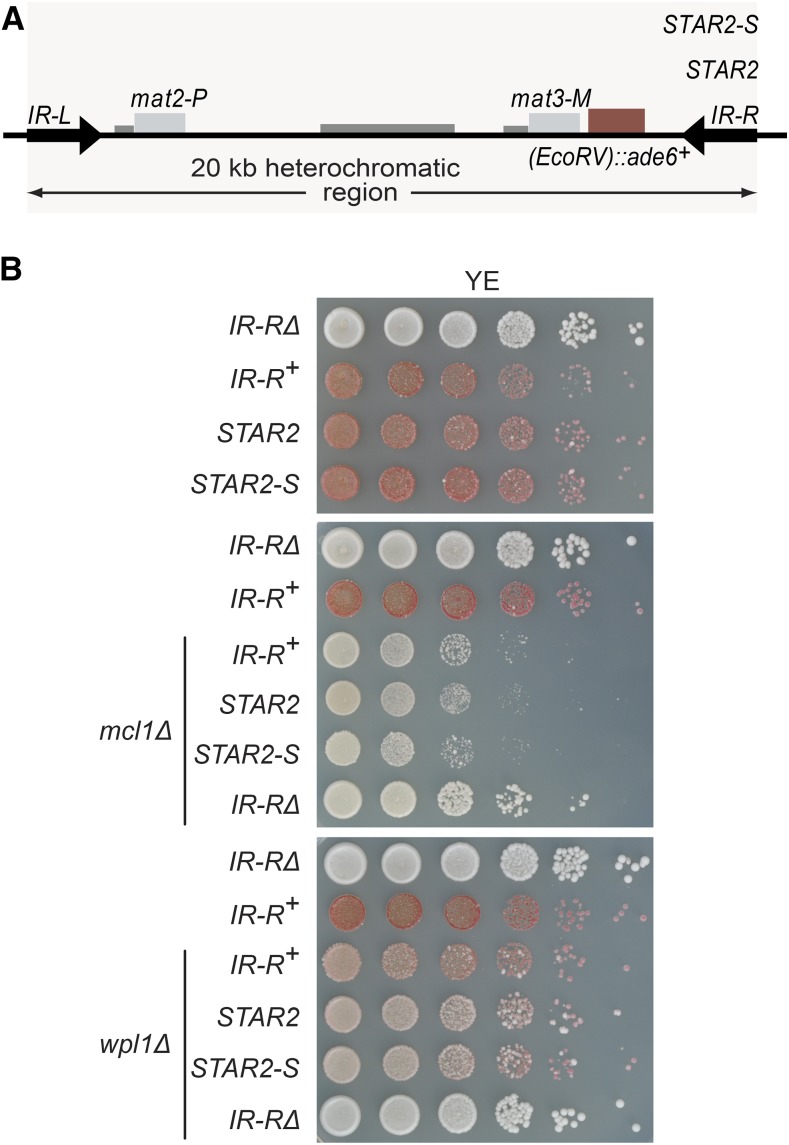
Effects of Mcl1 and Wpl1 on heterochromatic silencing. (A) The mating-type regions used in this experiment featured two ectopic boundaries, STAR2 and the 85 bp STAR2-S element (Toteva *et al.* 2017), in addition to wild-type *IR-R*^+^. (B) *mcl1*Δ and *wpl1*Δ mutants with the indicated boundaries were spotted on low adenine to compare *(EcoRV)*::*ade6*^+^ expression with the wild-type background. *mcl1*Δ confers a pronounced growth defect; however, the formed *(EcoRV)*::*ade6*^+^ *mcl1*Δ colonies are Ade^+^. *wpl1*Δ induces strong variegation. The strains were wild-type: PG3947, PG3950, PC152, and PT600; *mcl1*Δ: PA71, PA72, PA73, and PA74; and *wpl1*Δ: PA82, PA83, PA84, and PA85. YE, medium with limited adenine.

A few Bioneer strains could not be reliably retested by crossing. Instead, for three of them (*mcl1*Δ and *wpl1*Δ, scored as positive in first and second screens, and *dpb4*Δ, scored as false positive in first screen), the kanMX6 cassette that replaces each deleted ORF (Kim *et al.* 2010) was amplified by PCR, together with 500 bp–1 kb flanking DNA from genomic DNA of the Bioneer deletion strain, and used to transform tester strains to confirm effects suggested by the genetic screens. Little effect was seen in *dpb4*Δ cells, suggesting that the originally observed phenotype was artifactual, while derepression by *mcl1*Δ and *wpl1*Δ was confirmed (Figure 7).

The silencing factors that were retested throughout the whole scheme are listed in Table 1, together with references to prior literature for those that had been described previously. In addition to known components of heterochromatin, the screens identified factors that had eluded all previous screens or that had not been examined for effects in the mating-type region. A few were element-specific, such as Rif1 acting with the *STAR* elements (Toteva *et al.* 2017), while others were required for *(EcoRV)*::*ade6*^+^ silencing in all or nearly all query strains.

### Requirement for histone deacetylases and methyltransferases

The sole proteins required for *(EcoRV)*::*ade6*^+^ silencing in all configurations of the mating-type region tested were Clr1, Clr2, and Clr3; that is, the deacetylase module of the SHREC complex (Figure 2). Most boundary elements also relied on the chromatin remodeler module of SHREC, Mit1, with the exception of *BTH*1. Some independence of the deacetylase and remodeler modules of SHREC is predicted by a structure–function analysis of the complex (Job *et al.* 2016). The histone H3K9 methyltransferase Clr4, the Clr7, Clr8, and Rik1 subunits of the CLRC complex, and the chromodomain protein Swi6 were also broadly identified, in all screens except for the full-length rDNA repeat (Figure 2A). The rDNA repeat is exceptional in that it silences *(EcoRV)*::*ade6^+^* tightly through antisense transcription and histone deacetylation, even in the absence of Clr4 (Jakociunas *et al.* 2013). The Clr6 histone deacetylase-associated PHD protein-1 Cph1 was also identified in several screens, albeit with relatively weak phenotypes (Figure 2). Moreover, deletion of the *meu6^+^* gene adjacent to *clr4^+^* resulted in the same strong derepression as *clr4*Δ (Figure 2), possibly due to impaired *clr4* expression. Altogether, this first set of mutants emphasizes the central roles of deacetylation and methylation in heterochromatic gene silencing, and validates the screening strategy.

### Requirement for Ddb1-Cdt2 and Csn1

Nearly all screens also identified the Ddb1, Cdt2, and Csn1 proteins (Figure 2 and Figure S9 in File S1 for *ddb1*Δ). Ddb1 and Cdt2 are part of the Cullin 4 ubiquitin ligase CRL4^Cdt2^ that targets specific proteins for degradation by the proteasome. In Cullin complexes, an adaptor protein, Cdt2 in this case, interacts with cognate substrates to display them to the ubiquitin ligase activity. Three CRL4^Cdt2^ substrates are known in *S. pombe*: the putative histone H3K9 demethylase Epe1 (Braun *et al.* 2011), the licensing factor Cdt1 that is required for the firing of replication origins (Ralph *et al.* 2006); and the inhibitor of ribonucleotide reductase Spd1 (Liu *et al.* 2003). According to previous work, stabilization of Epe1 in the *cdt2*Δ and *ddb1*Δ mutants and its accumulation in heterochromatin would suffice to derepress *(EcoRV)*::*ade6*^+^ (Braun *et al.* 2011). Deletion of the COP9 signalosome (CSN) subunit Csn1 also resulted in reduced silencing (Figure 2). Reduced silencing in the *csn1*Δ mutant might result from CRL4^Cdt2^ deregulation (Liu *et al.* 2003), or from the destabilization of other cullin ligases such as SCF^Pof3^, whose F-box component Pof3 is proteolyzed in CSN mutants (Schmidt *et al.* 2009). Indicative of a general requirement in heterochromatin, Csn1 was also identified in a recent genetic screen for heterochromatic silencing defects at centromeres (Bayne *et al.* 2014).

### Requirement for Def1, Pof3, and Pli1

Further links to ubiquitylation were revealed with the identification of the proposed homolog of *S. cerevisiae* Def1 and the F-box protein Pof3 (Figure 3, Figure 4, Figure 5, Figure 6, and Figures S9 and S10 in File S1). To our knowledge, this is the first evidence for an effect of Def1 in heterochromatin. In *S. cerevisiae*, Def1 mediates the ubiquitylation and degradation of the Rpb1 subunit of RNA polymerase II (RNAPII) during transcriptional stress (Wilson *et al.* 2013), and of Pol3, the catalytic subunit of the replicative DNA polymerase δ, in postreplicative repair (Daraba *et al.* 2014). In *S. pombe*, Def1 might control polymerase usage in heterochromatin, remove RNAPII from heterochromatin to promote transcriptional silencing, or prevent collisions between DNA and RNA polymerases, a proposed cause of heterochromatin instability (Zaratiegui *et al.* 2011). The functions of Pof3, homologous to Dia2 in *S. cerevisiae*, are understood in much greater detail than those of Def1. In *S. pombe*, chromosomes are lost in the absence of Pof3, telomeres are shortened, telomere and centromere silencing is alleviated, and the DNA damage checkpoint is continuously activated (Katayama *et al.* 2002; Mamnun *et al.* 2006). Pof3 associates with the replisome. Its targets, inferred from the increased stability in *pof3*Δ cells, include the mediator of replication checkpoint Mrc1, Pol ε, and subunits of the CMG helicase, each of which could be relevant to the observed effects (see below; Roseaulin *et al.* 2013a,b; Maculins *et al.* 2015). Other targets of Pof3 potentially relevant to heterochromatic silencing would be Ams2, a regulator of histone gene transcription (Takayama *et al.* 2010), or other DNA-binding proteins that might be removed by Pof3 as Pof3 travels with the replication fork.

The screens also detected a small contribution of the SUMO E3 ligase Pli1 to *(EcoRV)*::*ade6^+^* silencing (Figure 3), compatible with previously observed limited derepression of centromeric reporters in *pli1*Δ cells (Xhemalce *et al.* 2004).

### Requirement for the RPC

Mrc1/Claspin, Mcl1 (Ctf4 in *S. cerevisiae*), Swi1 (Timeless in animals or Tof1 in *S. cerevisiae*), Swi3 (Tipin or Csm3), and the histone chaperone FACT are part of the RPC essential to normal replication fork progression (Gambus *et al.* 2006; Foltman *et al.* 2013; Yeeles *et al.* 2017). Here, Mrc1, Mcl1, Swi1, Swi3, and the Pob3 subunit of FACT were all necessary for tight *(EcoRV)*::*ade6^+^* silencing (Figure 4, Figure 7, and Figure S9 in File S1). Mrc1, Mcl1, Swi1, and Swi3 also ensure replication fork protection at replication blocks (Noguchi *et al.* 2004; Shimmoto *et al.* 2009) and telomeric repeats (Gadaleta *et al.* 2016). Structurally, Mcl1/Ctf4 forms a trimer that connects the CMG helicase to the lagging-strand primase Polα, and various accessory factors to the replisome (Williams and McIntosh 2002, 2005; Tsutsui *et al.* 2005; Simon *et al.* 2014; Samora *et al.* 2016; Villa *et al.* 2016). Pof3/Dia2 in particular, also identified in our screen, is associated with the replisome in an Mcl1- and Mrc1-dependent manner (Mamnun *et al.* 2006; Morohashi *et al.* 2009). Hence, the silencing defect in RPC mutants might be due to a failed association of Pof3 with the fork in these mutants. Since Pof3 is responsible for the short half-life of Mrc1 in wild-type *S. pombe* cells, and mediates the degradation of DNA polymerases and MCM helicases in the absence of the RPC (Roseaulin *et al.* 2013a,b), the silencing defect in *pof3*Δ cells might also result from a misregulated RPC.

### Further requirements for DNA replication/genome organization factors

Other proteins implicated in the control of DNA replication or genome organization identified in the screens were the forkhead protein Fkh2, whose *S. cerevisiae* homologs Fkh1 and Fkh2 organize the long-range clustering and activation of early-firing origins (Knott *et al.* 2012) and long-range chromosomal interactions occurring during mating-type switching (Li *et al.* 2012), and the cohesin release factor Wings apart-like Wpl1 (Figure 3, Figure 7, and Figure S9 in File S1). In the absence of Wpl1, *(EcoRV)*::*ade6^+^* showed a strongly variegated phenotype (Figure 7). In mammals, Δ*WAPL* cells display altered chromatin loop structure, reduced long-range chromosomal interactions in *cis*, and decreased nuclear compartmentalization that correlates with gene derepression in normally silent compartments (Haarhuis *et al.* 2017). These effects might result from the aberrant distribution or accumulation of cohesion, and might be evolutionarily conserved since cohesins participate in *S. pombe* genome architecture (Mizuguchi *et al.* 2014) and a *rad21* mutation causes heterochromatin loss in subtelomeric regions (Dheur *et al.* 2011). Furthermore, components of the RPC (including Swi1, Swi3, Mrc1, and Mcl1) are required for sister chromatid cohesion (*e.g.*, Williams and McIntosh 2002; Xu *et al.* 2004; Lengronne *et al.* 2006; Ansbach *et al.* 2008), hence the effects of the RPC, and Wpl1 might overlap mechanistically.

The screens also confirmed the importance of the iron–sulfur cluster assembly protein Mms19 to heterochromatin (Figure 3). Mms19 copurifies with the silencing factor Clr7/Dos2 (Li *et al.* 2011).

### Requirement for chromatin remodelers and chaperones

In addition to the SHREC subunit Mit1 and the FACT subunit Pob3, the SMARCAD1 family ATP-dependent DNA helicase Fft3 and the ATPase with bromodomain Abo1 were also broadly required for silencing by the various boundary elements used in the screens (Figure 3, Figure 4, Figure 5, Figure 6, and Figure S10 in File S1). In previous studies, Fft3 was found to affect heterochromatic boundaries at centromeres and telomeres (Stralfors *et al.* 2011), and functions of Abo1 in heterochromatin were recently documented as well (Gal *et al.* 2016). In our study, deletion of Abo1 conferred relatively modest, albeit distinguishable, phenotypes (Figure 3, Figure 4, Figure 5, Figure 6, and Figure S10 in File S1). The Abo1 paralogue Abo2 was not isolated in the initial screens, but was examined for comparison with Abo1 and displayed more minor effects, if any.

Newly identified factors whose deletion caused a pronounced derepression of *(EcoRV)*::*ade6^+^* included the predicted peptidyl-prolyl *cis*-trans isomerase Fkbp39 and the Ssz1 chaperone, an HSP70 family protein (Figure 3, Figure 4, and Figure 5 for effects on *(EcoRV)*::*ade6*^+^ with *IR-R^+^*; Figure S10 in File S1 for all elements; and Figure 6 for effects on *(EcoRV)*::*ura4*^+^). Both Fkbp39 and Ssz1 are predominantly nuclear in *S. pombe* (Matsuyama *et al.* 2006), which is compatible with them having effects on chromatin. The Fkbp39 homolog Fpr4 in *S. cerevisiae* catalyzes P30 and P38 isomerization in histone H3 (Nelson *et al.* 2006); in this light, our findings indicate a potential importance for this isomerization in heterochromatin.

### Requirement for other factors and modifying enzymes

Finally, Btf3, the Not2 and Rcd1 subunits of the CCR4-Not complex previously implicated in heterochromatic silencing in fission yeast (Cotobal *et al.* 2015; Bronner *et al.* 2017), and the histone acetyltransferase Gcn5 were also required, to various degrees, for silencing (Figure 3 and Figure S11 in File S1). Given the strong requirement for histone deacetylation in heterochromatin, a requirement for an opposing activity is intriguing, yet silent chromatin is not altogether deacetylated; Gcn5 might function in concert with Btf3 or Not2, or transient acetylation might facilitate heterochromatin maintenance through effects at the replication fork (Espinosa *et al.* 2010; Kurat *et al.* 2017).

### Effects at telomeres and centromeres

**Figure 8:**
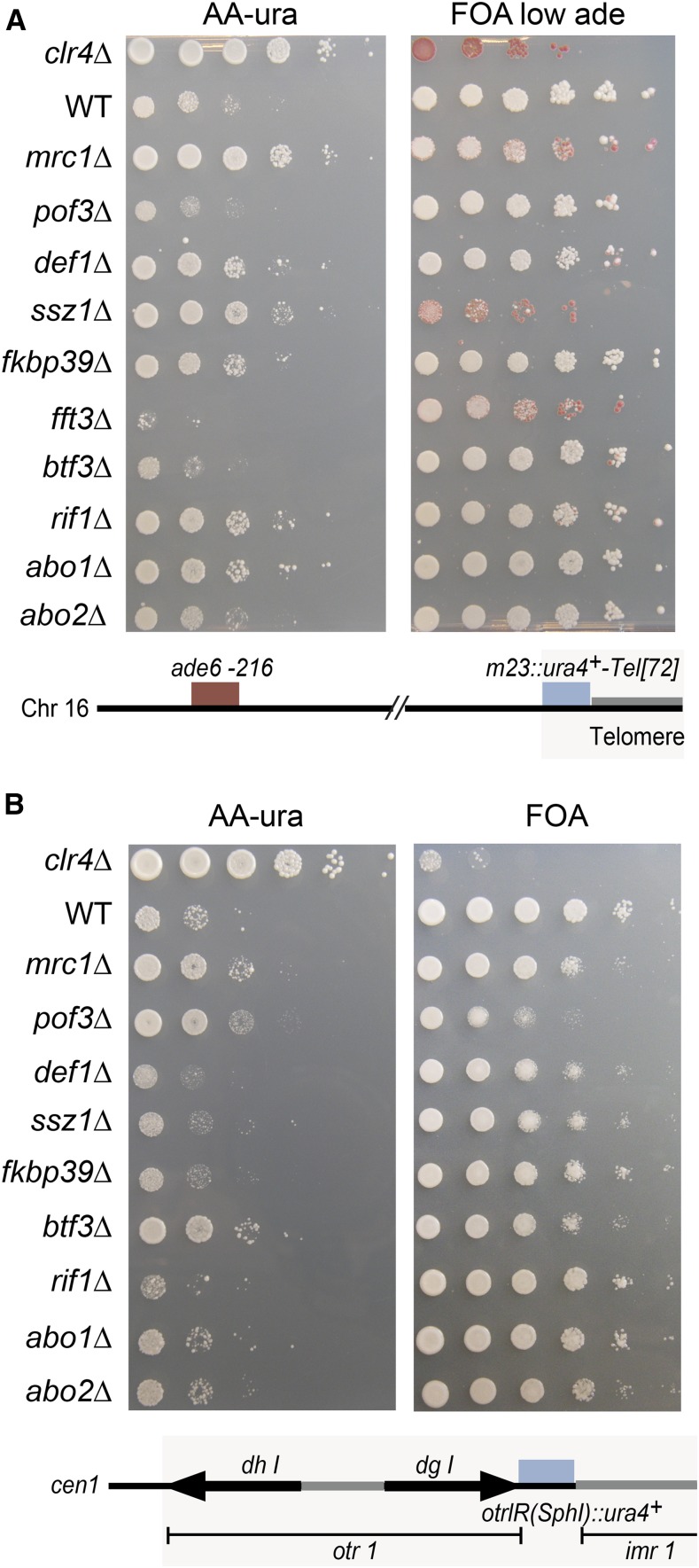
Effects of selected factors on telomeric and centromeric silencing. Serial dilutions (10-fold) of cell suspensions were spotted. (A) Effects on the *m23*::*ura4^+^-Tel[72]* telomeric reporter gene. Derepression of the reporter improves plating efficiency on AA-ura and decreases white colony formation on FOA medium with low adenine concentration (where only cells with a repressed reporter can form white colonies). Strains used: LJ171, FY520, LJ163, LJ172, LJ173, LJ162, LJ174, LJ165, LJ170, LJ168, LJ161, and LJ166. (B) Effects on centromeric *otr1(SphI)*::*ura4^+^* reporter gene. Strains used: LJ185, FY648, LJ180, LJ186, LJ190, LJ192, LJ187, LJ183, LJ181, LJ191, and LJ189. AA-ura, medium lacking uracil; ade, adenine; Chr, chromosome; FOA, fluoroorotic acid; WT, wild-type.

The effects of selected ORF deletions on telomeric silencing was assayed with the m23::*ura4*^+^-Tel[72] reporter close to the end of a truncated minichromosome Ch16 ((Nimmo *et al.* 1994); Figure 8A). In this assay, cells containing the minichromosome are Ade^+^ due to intragenic complementation between the *ade6-210* allele on chromosome 3 and *ade6-216* on the minichromosome. Several deletions (*mrc1*Δ, *def1*Δ, *ssz1*Δ, *fkbp39*Δ, *rif1*Δ, and *rif1*Δ) increased growth on medium lacking uracil, indicative of m23::*ura4*^+^-Tel[72] derepression. For some strains (*mrc1*Δ, *ssz1*Δ, and *fft3*Δ in Figure 8A), FOA strongly selected for cells that had lost their minichromosome, resulting in red colonies on FOA with limiting adenine. This is expected for mutants in which *ura4*^+^ cannot be repressed effectively; only cells that have lost the mini-chromosome are phenotypically Ura^−^.

The *otr1R(SphI)*::*ura4*^+^ insertion in centromere 1 (Allshire *et al.* 1995) was used to test effects on pericentromeric silencing. These were less pronounced than for the edge of the mating-type region or subtelomere (Figure 8B), perhaps due to the location of the *otr1R(SphI)*::*ura4*^+^ reporter, embedded in RNAi-responsive heterochromatin.

### Interplays between replication and heterochromatin

**Figure 9:**
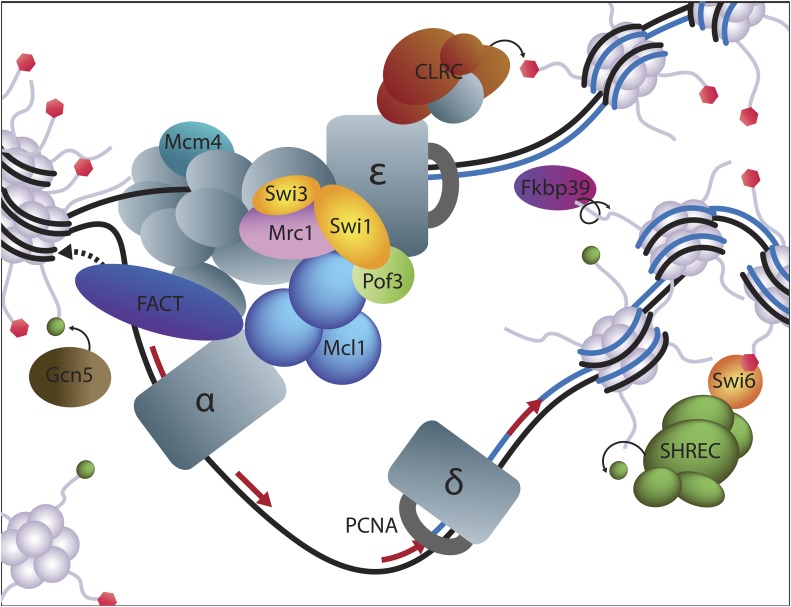
Silencing factors identified in this screen have roles in DNA replication. The drawing schematizes a replication fork. Factors identified in the screen are in color, while other subunits of the represented complexes are in gray.

Physical and functional links between DNA replication and heterochromatin formation have been discovered in numerous organisms, and are proposed to participate in the epigenetic maintenance of heterochromatin during cell division. In *S. pombe*, The Clr7/Dos2 subunit of the H3K9 methyltransferase complex CLRC binds Polε, and Polε mutants with reduced affinity for Dos2/Clr7 exhibit heterochromatin defects (Li *et al.* 2011). Mutations in the primase Polα (Nakayama *et al.* 2001; Natsume *et al.* 2008) or in the Mcm4 helicase (Toteva *et al.* 2017) also cause heterochromatin defects, as well as mutations in nonessential replication factors (Natsume *et al.* 2008). Our present results emphasize the tight links between replication and heterochromatin integrity by identifying multiple RPC components and associated chromatin modifiers (Figure 9).

A first mechanism through which RPC components might facilitate the maintenance of heterochromatin is through the stabilization of ongoing replication forks. It has been observed that replisome components are, under some circumstances, degraded upon replication stress (Roseaulin *et al.* 2013a,b; Daraba *et al.* 2014); in particular, both Polε and replicative helicases become unstable in *S. pombe* in the absence of the RPC component Swi1 (Roseaulin *et al.* 2013a,b). The breakdown of replisomes at replication forks that encounter an impediment in heterochromatin might lead to the dissociation of CLRC from the fork, or to a fork restart using different DNA polymerases that do not interact with CLRC, thereby weakening heterochromatin (Zaratiegui *et al.* 2011; Lee *et al.* 2013; Lee and Russell 2013). Similarly, control of homologous recombination, both during and outside S phase, might favor heterochromatin maintenance by preventing the use of polymerases other than Polε (Zaratiegui *et al.* 2011; Miyabe *et al.* 2015). A requirement for the stabilization of replication forks in heterochromatic regions would be compounded by the fact that heterochromatin appears to be difficult to replicate and prone to fork stalling (Zaratiegui *et al.* 2011; Lee *et al.* 2013; Lee and Russell 2013).

A second mechanism through which RPC components might reinforce heterochromatin is through the control of replication origin firing. Mrc1/Claspin has dual roles in replication fork progression and in the DNA replication timing program. Other factors isolated in our screen also affect replication origin usage. Thus Rif1, necessary for boundary activity and heterochromatin protection by the *STAR* boundaries (Toteva *et al.* 2017), controls the replication timing program across species (Hayano *et al.* 2012; Yamazaki *et al.* 2012; Peace *et al.* 2014). In *S. pombe*, both Mrc1 and Rif1 mutations alter DNA replication genome-wide and bypass the requirement for the DDK kinase Hsk1 (Matsumoto *et al.* 2011; Hayano *et al.* 2012). Consistent with coordinated control of origin firing and heterochromatin maintenance, a mutation in the N-terminus of the Mcm4 helicase, which removes a part of the protein that is predicted to confer DDK control on origin firing, weakens heterochromatic silencing close to *IR-R*^+^ or artificial boundaries (Toteva *et al.* 2017). In addition, the forkhead protein Fkh2 has well-documented roles in replication origin usage in *S. cerevisiae* (Knott *et al.* 2012). Altogether, these observations indicate that the separation of replication domains, possibly facilitated by boundary elements, is of importance to heterochromatin integrity, for instance by controlling the type of replisomes that operate in particular domains.

## Supplementary Material

Supplemental material is available online at www.g3journal.org/lookup/suppl/doi:10.1534/g3.117.300341/-/DC1.
