# Supplementary material for: Dependency of Heterochromatin Domains on Replication Factors
Source: G3 (Bethesda). 2017 Nov 29;8(2):477–89. doi: 10.1534/g3.117.300341 (PMC5919735; doi:10.1534/g3.117.300341)
Supplement: Supplementary file 1 [file 477FileS1.pdf]

SUPPLEMENTAL MATERIAL

Table S1 presents a list of Bioneer deletion strains selected for the second and third mutant screens and Table S2 a list of potential false positives. Figure S1 to S8 show the phenotypes of mutants examined in the second screen; Figure S9 to S11 show the phenotypes of mutants examined in the third screen. Table S3 contains a list of strains and their genotypes; Table S4 contains oligonucleotide sequences.

Table S1. Bioneer deletion strains selected for second and third screens. óPlate positionô refers to the plate images in Figure S1-8 showing the results of the second screen.

Deletion strains used in 2nd screen				used in 3rd screen	Final list
Plate position	Bioneer #	ID	Gene description		
A1	V3-P01-05	SPAC11E3.15	60S ribosomal protein L22 (predicted)		
A2	V3-P01-17	SPAC1687.23c	sequence orphan		
A3	V3-P02-16	SPAPJ691.03	mitochondrial conserved eukaryotic protein		
A4	V3-P02-40	SPBC1711.01c	mating-type m-specific polypeptide mi 1		
A5	V3-P02-41	SPBC1711.02	mating-type m-specific polypeptide mc 1		
A6	V3-P02-75	SPBC4B4.11	conserved fungal protein		
A7	V3-P03-81	SPAC1687.05	SUMO E3 ligase Pli1	•	•
A8	V3-P03-83	SPAC1687.15	serine/threonine protein kinase Gsk3		
A9	V3-P04-23	SPAC1851.03	CK2 family regulatory subunit Ckb1	•	•
A10	V3-P04-49	SPAC227.10	prefoldin subunit 2 (predicted)		
A11	V3-P05-23	SPAC2F7.09c	mitochondrial GTPase related protein (predicted)	•	
A12	V3-P05-24	SPAC2F7.10	palmitoyltransferase (predicted)		
A13	V3-P05-33	SPAC31G5.19	ATPase with bromodomain protein Abo1	•	•
A14	V3-P05-52	SPAC3C7.10	peroxin 13 (predicted)		
A15	V3-P05-57	SPAC3G6.04	RNA-binding protein Rnp24		
A16	V3-P05-67	SPAC3H5.12c	60S ribosomal protein L5 (predicted)		
B1	V3-P06-02	SPAC57A7.12	heat shock protein Ssz1(predicted)	•	•
B2	V3-P06-05	SPAC5D6.06c	UDP-GlcNAc transferase associated protein Alg14 (predicted)		
B3	V3-P06-14	SPAC664.01c	chromodomain protein Swi6	•	•
B4	V3-P06-19	SPAC694.06c	mediator of replication checkpoint 1	•	•
B5	V3-P06-41	SPAC824.08	guanosine-diphosphatase Gda1		
B6	V3-P06-95	SPBC12C2.02c	Rictor homolog, Ste20		
B7	V3-P07-42	SPBC1709.14	peptide N-glycanase (predicted)		
B8	V3-P07-44	SPBC1718.06	mitochondrial dynamin family GTPase Msp1		
B9	V3-P07-46	SPBC1734.06	Rad18 homolog Rhp18	•	
B10	V3-P07-80	SPBC19G7.10c	topoisomerase II-associated deadenylation-dependent mRNA-decapping factor Pdc2 (predicted)	•	
B11	V3-P08-10	SPBC23E6.08	Golgi membrane exchange factor subunit Sat1 (predicted)	•	
B12	V3-P08-53	SPBC336.14c	serine/threonine protein kinase, PAN complex subunit, Ppk26		
B13	V3-P08-63	SPBC365.04c	RNA-binding protein, involved in ribosome biogenesis (predicted)	•	

B14	V3-P09-27	SPBP16F5.05c	ribosome biogenesis protein Nop8 (predicted)		
B15	V3-P09-30	SPBP22H7.05c	ATPase with bromodomain protein Abo2 (predicted)	•[a]	•[a]
B16	V3-P09-32	SPBP35G2.10	SHREC complex subunit Mit1	•	•
C1	V3-P09-52	SPCC1183.02	glutathione S-transferase (predicted)		
C2	V3-P09-54	SPCC11E10.08	silencing protein Rik1	•	•
C3	V3-P09-69	SPCC1450.11c	serine/threonine protein kinase Cek1		
C4	V3-P10-07	SPCC553.08c	GTPase Ria1 (predicted)		
C5	V3-P10-23	SPCC74.06	histidine kinase Mak3		
C6	V3-P10-35	SPAC10F6.11c	autophagy associated protein kinase activator Atg17		
C7	V3-P10-52	SPAC17H9.10c	damaged DNA binding protein Ddb1	•	•
C8	V3-P10-53	SPAC1805.10	sequence orphan		
C9	V3-P11-19	SPAPYUG7.03c	medial ring protein Mid2		
C10	V3-P11-68	SPBC800.03	histone deacetylase (class II) Clr3	•	•
C11	V3-P11-94	SPCC338.16	F-box protein Pof3	•	•
C12	V3-P12-13	SPCPB1C11.03	cysteine transporter (predicted)		
C13	V3-P12-29	SPAC13A11.06	pyruvate decarboxylase (predicted)		
C14	V3-P12-43	SPAC1B2.03c	GNS1/SUR4 family protein (predicted)		
C15	V3-P12-46	SPAC1B3.17	chromatin silencing protein Clr2	•	•
C16	V3-P12-69	SPAC25A8.02	sequence orphan		
D1	V3-P12-78	SPAC30D11.05	AP-3 adaptor complex subunit Aps3 (predicted)		
D2	V3-P13-41	SPBC18E5.01	cycloisomerase 2 family		
D3	V3-P14-03	SPCC11E10.05c	nucleoside diphosphatase Ynd1	•	
D4	V3-P14-10	SPCC1322.02	sequence orphan		
D5	V3-P14-25	SPCC188.07	telomere maintenance protein Ccq1	•	
D6	V3-P14-50	SPAC11G7.01	sequence orphan		
D7	V3-P14-52	SPAC12G12.09	conserved fungal protein		
D8	V3-P14-53	SPAC13F5.04c	endosomal sorting protein (predicted)		
D9	V3-P14-69	SPAC17H9.19c	WD repeat protein Cdt2	•	•
D10	V3-P14-80	SPAC22F8.07c	replication termination factor Rtf1	•	
D11	V3-P15-44	SPBC14F5.03c	karyopherin Kap123		
D12	V3-P15-63	SPBC2D10.17	cryptic loci regulator Clr1	•	•
D13	V3-P15-77	SPBC428.08c	histone H3 methyltransferase Clr4	•	•
D14	V3-P15-80	SPBC4F6.11c	asparagine synthase (predicted)		
D15	V3-P16-15	SPCC306.11	sequence orphan		
D16	V3-P16-20	SPCC4G3.15c	CCR4-Not complex subunit Not2 (predicted)	•	•
E1	V3-P16-29	SPCC965.05c	uracil DNA N-glycosylase Thp1	•	
E2	V3-P16-66	SPAC31G5.09c	MAP kinase Spk1		
E3	V3-P17-85	SPCC757.09c	RNA-binding protein that suppresses calcineurin deletion Rnc1	•	

E4	V3-P18-15	SPAC19D5.02c	peroxisomal membrane protein Pex22 (predicted)		
E5	V3-P18-50	SPAPB24D3.08c	NADP-dependent oxidoreductase (predicted)		
E6	V3-P18-68	SPBC28F2.02	mRNA export protein Mep33		
E7	V3-P18-94	SPCC1442.07c	ubiquitin/metalloprotease fusion protein		
E8	V3-P19-39	SPAC18G6.04c	serine hydroxymethyltransferase Shm2 (predicted)		
E9	V3-P19-50	SPAC24C9.12c	glycine hydroxymethyltransferase (predicted)	•	
E10	V3-P19-56	SPAC30C2.07	sequence orphan		
E11	V3-P19-82	SPBC14F5.11c	sorting nexin Snx41 (predicted)		
E12	V3-P20-37	SPCC70.10	sequence orphan		
E13	V3-P20-41	SPAC1002.12c	succinate-semialdehyde dehydrogenase (predicted)		
E14	V3-P20-59	SPAC21E11.03c	transcription factor Pcr1		
E15	V3-P20-82	SPAPYUG7.04c	DNA-directed RNA polymerase II complex subunit Rpb9		
E16	V3-P21-16	SPBC83.09c	GYF domain protein		
F1	V3-P21-34	SPCC569.02c	S. pombe specific UPF0321 family protein 2		
F2	V3-P21-50	SPAC1F7.10	hydantoin racemase family (predicted)		
F3	V3-P21-57	SPAC29B12.06c	RNA-binding protein, CCR4-NOT complex subunit Rcd1	•	•
F4	V3-P21-63	SPAC589.10c	ribosomal-ubiquitin fusion protein Ubi5 (predicted)		
F5	V3-P21-64	SPAC5H10.02c	ThiJ domain protein		
F6	V3-P21-90	SPBC2G5.01	DUF1682 family protein		
F7	V3-P22-54	SPBC215.03c	COP9/signalosome complex subunit Csn1	•	•
F8	V3-P23-38	SPBC106.01	dual specificity protein kinase Mph1		
F9	V3-P23-49	SPBC36B7.06c	sequence orphan		
F10	V3-P23-58	SPCC613.11c	cell surface glycoprotein (predicted), DUF1773 family protein 2		
F11	V3-P23-75	SPAPB8E5.10	conserved fungal protein, with meiosis specific splicing		
F12	V3-P23-80	SPBC3B8.10c	Nem1-Spo7 phosphatase complex catalytic subunit Nem1 (predicted)		
F13	V3-P24-07	SPAC869.07c	alpha-galactosidase, melibiase		
F14	V3-P24-57	SPAC1687.07	conserved fungal protein		
F15	V3-P24-62	SPAP27G11.06c	AP-1 adaptor complex sigma subunit Aps1		
F16	V3-P24-80	SPAC7D4.08	sequence orphan		
G1	V3-P25-45	SPAC15E1.07c	meiotic cohesin complex associated protein Moa1		
G2	V3-P25-50	SPAC2E1P5.02c	Rab GTPase binding protein upregulated in meiosis II (predicted)		
G3	V3-P25-57	SPBC365.11	GRIP domain protein		
G4	V3-P25-58	SPBC4B4.03	RSC complex subunit Rsc1		

G5	V3-P25-74	SPBC21C3.19	SBDS family protein Rtc3 (predicted)		
G6	V3-P25-91	SPBPJ4664.05	conserved fungal protein		
G7	V3-P25-93	SPCPJ732.01	retromer complex subunit Vps5		
G8	V3-P25-94	SPAC1142.01	DUF654 family protein		
G9	V3-P25-95	SPAC15A10.15	shugoshin Sgo2		
G10	V3-P26-29	SPAC29A4.05	myosin I light chain Cam2		
G11	V3-P26-47	SPBC428.07	meiotic chromosome segregation protein Meu6	•	•
G12	V3-P26-71	SPCC594.07c	bouquet formation protein Bqt3	•	
G13	V3-P26-77	SPAC2E1P3.01	dehydrogenase (predicted)		
G14	V3-P26-91	SPBC16A3.12c	triglyceride lipase-cholesterol esterase (predicted)		
G15	V3-P26-94	SPBC609.05	FACT complex component Pob3	•	•
G16	V3-P27-16	SPBP8B7.21	ubiquitin C-terminal hydrolase Ubp3		
H1	V3-P27-23	SPAC26A3.02	adenine DNA glycosylase Myh1		
H2	V3-P27-24	SPAC2G11.06	AAA family ATPase Vps4 (predicted)		
H3	V3-P27-26	SPAC6C3.03c	sequence orphan		
H4	V3-P27-29	SPAC926.02	conserved fungal protein		
H5	V3-P27-31	SPBC21B10.06c	myosin binding vezatin family protein involved in peroxisome inheritance Inp2 (predicted)		
H6	V3-P27-38	SPAC11H11.01	ESCRT I complex subunit Vps23		
H7	V3-P27-53	SPBC6B1.09c	Mre11 complex subunit Nbs1		
H8	V3-P27-56	SPAC31F12.01	zds family protein phosphatase type A regulator Zds1 (predicted)		
H9	V3-P27-64	SPAC140.03	argonaute binding protein 1		
H10	V3-P27-69	SPBC83.11	ER triose phosphate transmembrane transporter (predicted)		
H11	V3-P27-95	SPBC1289.11	splicing factor Spf38		
H12	V3-P28-23	SPCC794.02	wtf element Wtf5		
H13	V3-P28-34	SPAC29E6.07	sequence orphan		
H14	V3-P28-69	SPAC1687.21	phosphoglycerate mutase family (predicted)		
H15	V3-P28-70	SPAC6F6.17	telomere length regulator protein Rif1	•	•
H16	V3-P28-71	SPBC800.02	cell cycle transcriptional repressor Whi5 (predicted)		
I1	V3-P28-91	SPBC365.20c	nicotinamidase (predicted)		
I2	V3-P29-18	SPBC13G1.12	vacuolar sorting protein Did2 (predicted)	•	
I3	V3-P29-20	SPBC17D11.02c	ubiquitin-protein ligase Hrd1, synviolin family		
I4	V3-P29-23	SPBC1A4.03c	DNA topoisomerase II		
I5	V3-P29-39	SPAC4G9.19	DNAJ domain protein DNAJB family (predicted)		
I6	V3-P29-55	SPCC1223.15c	DASH complex subunit Spc19	•	
I7	V3-P29-74	SPAC16A10.01	DUF1212 family protein		

I8	V3-P29-75	SPAC16C9.05	Clr6 histone deacetylase associated PHD protein-1 Cph1	•	•
I9	V3-P29-80	SPAC1952.16	RhoGAP, GTPase activator towards Rho/Rac/Cdc42-like small GTPases (predicted)		
I10	V3-P29-89	SPAC19D5.03	poly(A) polymerase Cid1		
I11	V3-P30-11	SPAC25A8.01c	fun thirty related protein Fft3	•	•
I12	V3-P30-31	SPAC607.06c	metallopeptidase		
I13	V3-P30-41	SPAPB24D3.09c	ABC transporter Pdr1		
I14	V3-P30-46	SPBC1347.02	FKBP-type peptidyl-prolyl cis-trans isomerase (predicted)	•	•
I15	V3-P30-81	SPBC3B8.06	conserved fungal protein		
I16	V3-P30-86	SPBC530.01	GTPase activating protein Gyp1 (predicted)		
J1	V3-P30-89	SPBC557.04	Ark1/Prk1 family protein kinase Ppk29		
J2	V3-P31-01	SPBC725.06c	serine/threonine protein kinase Ppk31 (predicted)		
J3	V3-P31-18	SPCC1322.14c	vacuolar transporter chaperone (VTC) complex subunit (predicted)		
J4	V3-P31-22	SPCC162.10	serine/threonine protein kinase Ppk33 (predicted)		
J5	V3-P31-44	SPCC965.06	potassium channel subunit (predicted)		
J6	V3-P31-47	SPCC970.07c	Rik1-associated factor Raf2	•	•
J7	V3-P31-64	SPAC24H6.03	cullin 3		
J8	V3-P31-66	SPAC323.07c	MatE family transporter (predicted)		
J9	V3-P31-75	SPBC25D12.05	N2,N2-dimethylguanosine tRNA methyltransferase		
J10	V3-P31-78	SPBC359.03c	amino acid transporter Aat1 (predicted)		
J11	V3-P31-83	SPCC1322.12c	serine/threonine protein kinase Bub1		
J12	V3-P31-89	SPCC285.16c	MutS protein homolog	•	
J13	V3-P31-92	SPCC584.11c	Svf1 family protein Svf1		
J14	V3-P32-21	SPAC977.16c	dihydroxyacetone kinase Dak2		
J15	V3-P32-46	SPCC4B3.03c	mitochondrial morphology protein (predicted)		
J16	V3-P32-63	SPAC3G6.06c	FEN-1 endonuclease Rad2		
K1	V3-P32-77	SPBC428.17c	Wings apart-like homolog Wpl1	•	•
K2	V3-P32-78	SPBC4F6.16c	ER oxidoreductin Ero1a		
K3	V3-P33-11	SPAC2E1P3.02c	ammonium transporter Amt3		
K4	V3-P33-12	SPAC30D11.07	DNA endonuclease III		
K5	V3-P33-28	SPBC646.02	complexed with Cdc5 protein Cwf11		
K6	V3-P33-45	SPAC1F8.03c	siderophore-iron transporter Str3		
K7	V3-P33-49	SPAC23C11.06c	hydrolase (inferred from context)		
K8	V3-P33-53	SPAC869.06c	HHE domain cation binding protein		
K9	V3-P33-61	SPBC16G5.15c	fork head transcription factor Fkh2	•	•
K10	V3-P33-72	SPCC16A11.03c	DUF2009 protein		

K11	V3-P33-82	SPCC794.09c	translation elongation factor EF-1 alpha Ef1a-a		
K12	V3-P33-84	SPCPB16A4.02c	conserved fungal protein		
K13	V3-P34-37	SPAC4F10.14c	Basic transcription factor Btf3	•	•
K14	V3-P34-51	SPAC4G9.12	gluconokinase		
K15	V3-P34-60	SPBC3H7.05c	sequence orphan		
K16	V3-P34-61	SPBC3H7.14	BRCT domain protein		
L1	V3-P34-86	SPBC3E7.02c	heat shock protein Hsp16		
L2	V3-P34-90	SPCC417.02	DASH complex subunit Dad5		
L3	V3-P35-03	SPAC3H1.08c	DUF1640 family protein		
L4	V3-P35-05	SPBC1539.07c	glutathione-dependent formaldehyde dehydrogenase (predicted)		
L5	V3-P35-25	SPAC27D7.04	4-alpha-hydroxytetrahydrobiopterin dehydratase (predicted)		
L6	V3-P35-44	SPCC1020.13c	DDHD family phospholipase (predicted)		
L7	V3-P35-48	SPAC13G6.13	sequence orphan		
L8	V3-P35-62	SPBC16G5.09	serine carboxypeptidase (predicted)		
L9	V3-P35-81	SPAC12G12.11c	DUF544 family protein		
L10	V3-P35-84	SPCC4B3.05c	uroporphyrinogen decarboxylase Hem12 (predicted)		
L11	V3-P36-01	SPCC613.08	CDK regulator, involved in ribosome export (predicted)		
L12	V3-P36-27	SPBC1198.09	ubiquitin conjugating enzyme Ubc16 (predicted)		
L13	V3-P36-35	SPAC3H1.07	arginase Aru1		
L14	V3-P36-44	SPBC3E7.06c	vacuolar membrane amino acid uptake transporter Fnx2		
L15	V3-P36-54	SPCC364.01	calnexin independence factor Cif1		
Deletion strains independently re-tested and added to final list					
-	V3-P11-59	SPBC354.10	RNAPII degradation factor Def1 (predicted)	•	•
-	V3-P19-76	SPAPB1E7.02c	DNA polymerase alpha accessory factor Mcl1	•	•
-	V3-P20-56	SPAC1952.05	SAGA complex histone acetyltransferase catalytic subunit Gcn5	•	•
-	V3-P30-44	SPBC1198.11c	RNA polymerase I transcription termination factor Reb1	•	•
-	V3-P03-26	SPAC1071.02	Dos2 silencing complex subunit Mms19	•	•
-	added[b]	SPBC216.06c	replication fork protection complex subunit Swi1	•	•
-	added[b]	SPBC30D10.04	replication fork protection complex subunit Swi3	•	•
-	added[b]	SPCC613.12c	CLRC ubiquitin E3 ligase complex specificiy factor Clr8/Raf1/Dos1	•	•

[a] Abo2 was examined for comparison with Abo1.
[b] These strains were not in the V3 Bioneer library.

Table S2. Potential false positives. The strains listed in this Table produced Ade$^+$ colonies in the absence of crossing indicating the Ade$^+$ phenotype following crosses is not likely to reflect *(EcoRV)::ade6*$^+$expression.

V3-P02-69	SPBC3D6.09	DNA polymerase epsilon subunit Dpb4
V3-P11-45	SPBC23E6.09	transcriptional co-repressor Ssn6
V3-P15-60	SPBC25D12.02c	nucleolar protein Dnt1
V3-P17-26	SPBC1A4.09	pseudouridine synthase (predicted)
V3-P17-46	SPBC577.05c	meiotic recombination protein Rec27
V3-P17-49	SPBC776.06c	Arf3/6 docking factor (predicted)
V3-P19-93	SPBC216.03	conserved fungal protein
V3-P19-94	SPBC21C3.12c	DUF953 family protein
V3-P20-13	SPBC56F2.01	F-box protein Pof12
V3-P21-03	SPBC21.03c	DUF55 family protein
V3-P21-93	SPBC582.06c	horsetail movement protein Hrs1/Mcp6
V3-P23-51	SPBC887.06c	sorting nexin Snx3 (predicted)
V3-P28-66	SPBC16E9.18	phosphatidylserine decarboxylase Psd1
V3-P28-92	SPBC13G1.14c	RNA-binding protein (predicted)
V3-P29-55	SPCC1223.15c	DASH complex subunit Spc19
V3-P34-57	SPBC21C3.07c	actin binding methyltransferase (predicted)
V3-P35-71	SPBC21.02	TLDc domain protein 2

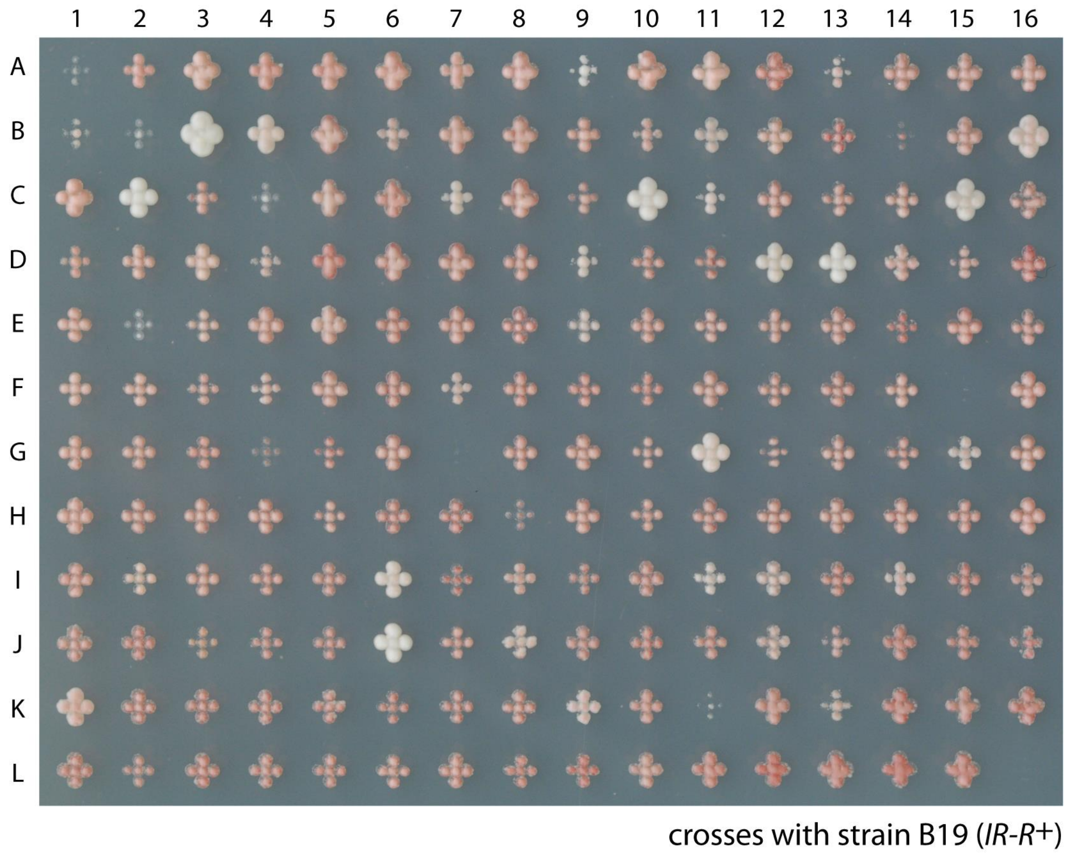

Figure S1. Second screen with *IR-R*$^{+}$boundary. Strain B19 was crossed with strains from the Bioneer collection as described. *(EcoRV)::ade6*$^{+}$expression was assayed in progeny combining *(EcoRV)::ade6*$^{+}$*IR-R*$^{+}$with each ORF deletion listed in Table S1. In the image shown, the recombinant progeny was propagated on NBA medium supplemented with leucine to assess *(EcoRV)::ade6*$^{+}$expression.

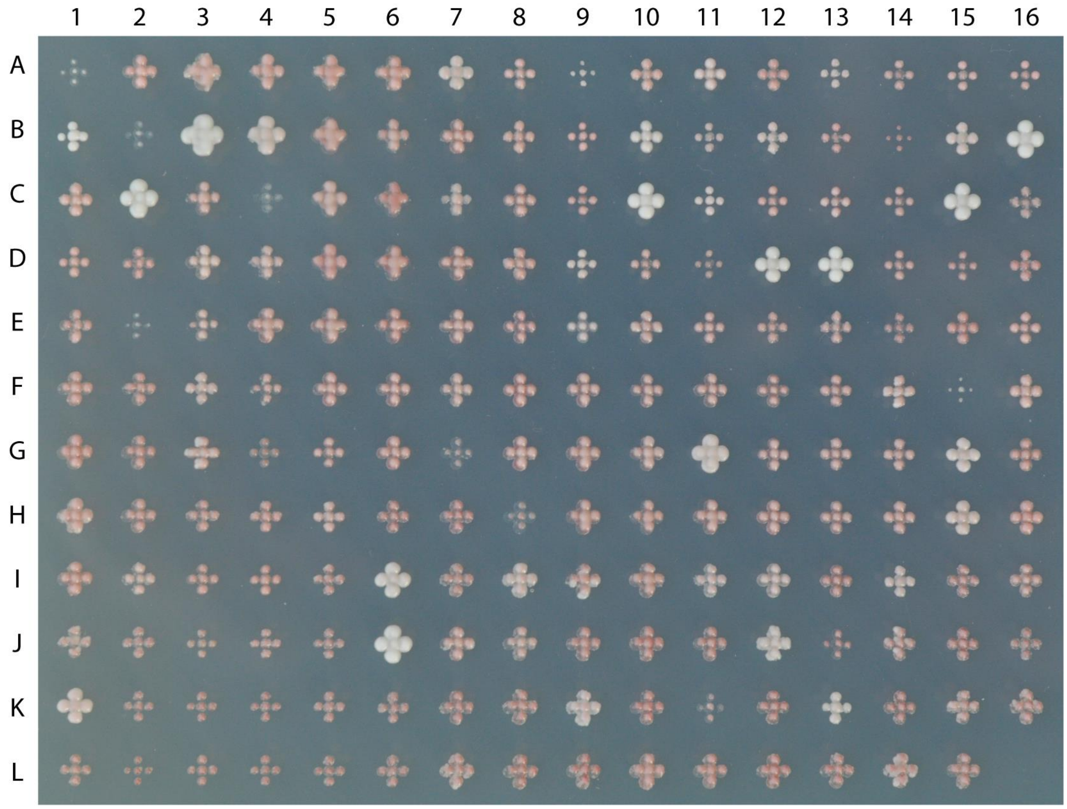

Figure S2. Second screen with *STAR1* boundary. Same as Figure S1, with strain B15 in which *IR-R*$^{+}$is replaced with the *STAR1* element.

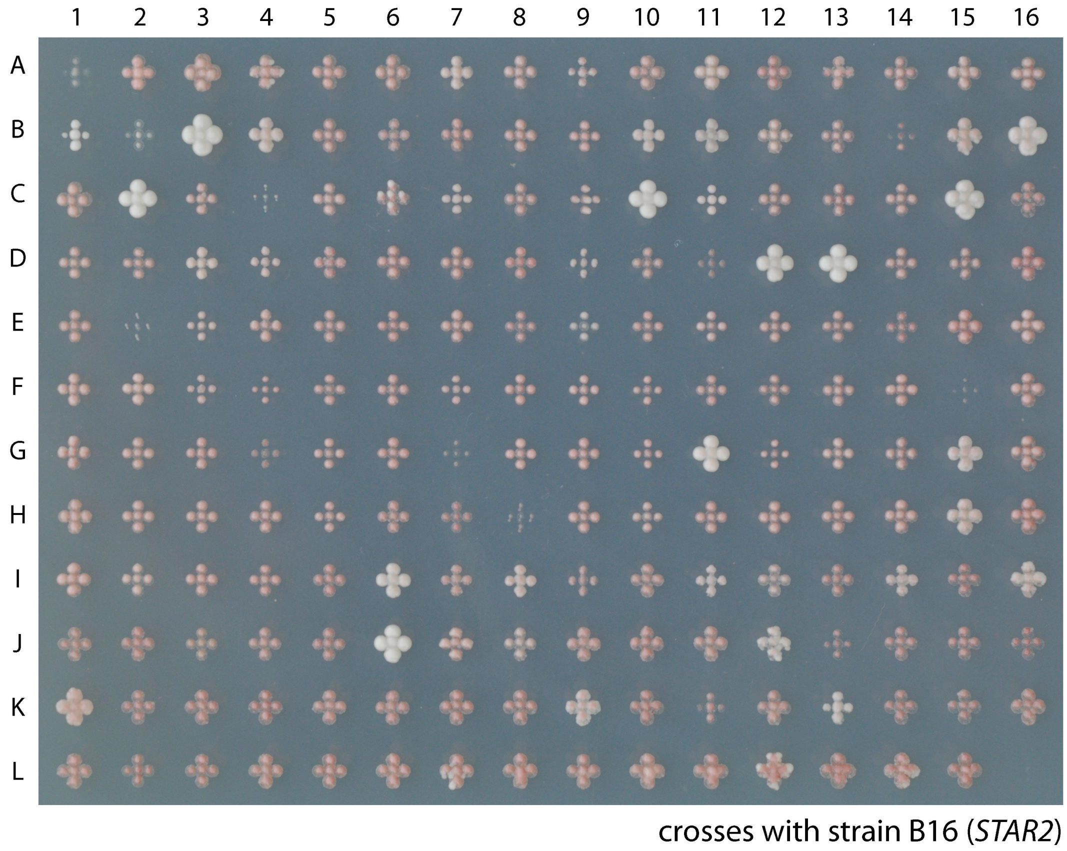

Figure S3. Second screen with *STAR2* boundary. Same as Figure S1, with strain B16 in which *IR-R*$^{+}$is replaced with the *STAR2* element.

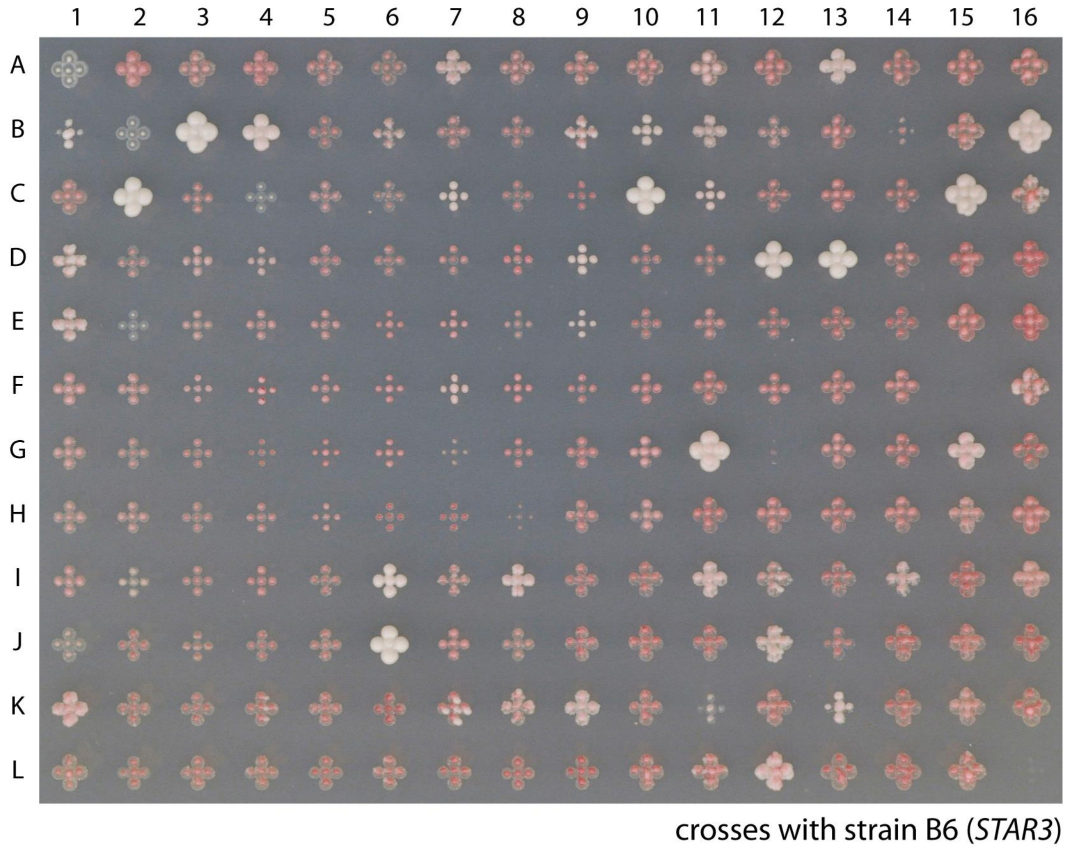

Figure S4. Second screen with *STAR3* boundary. Same as Figure S1, with strain B6 in which *IR-R*$^{+}$is replaced with the *STAR3* element.

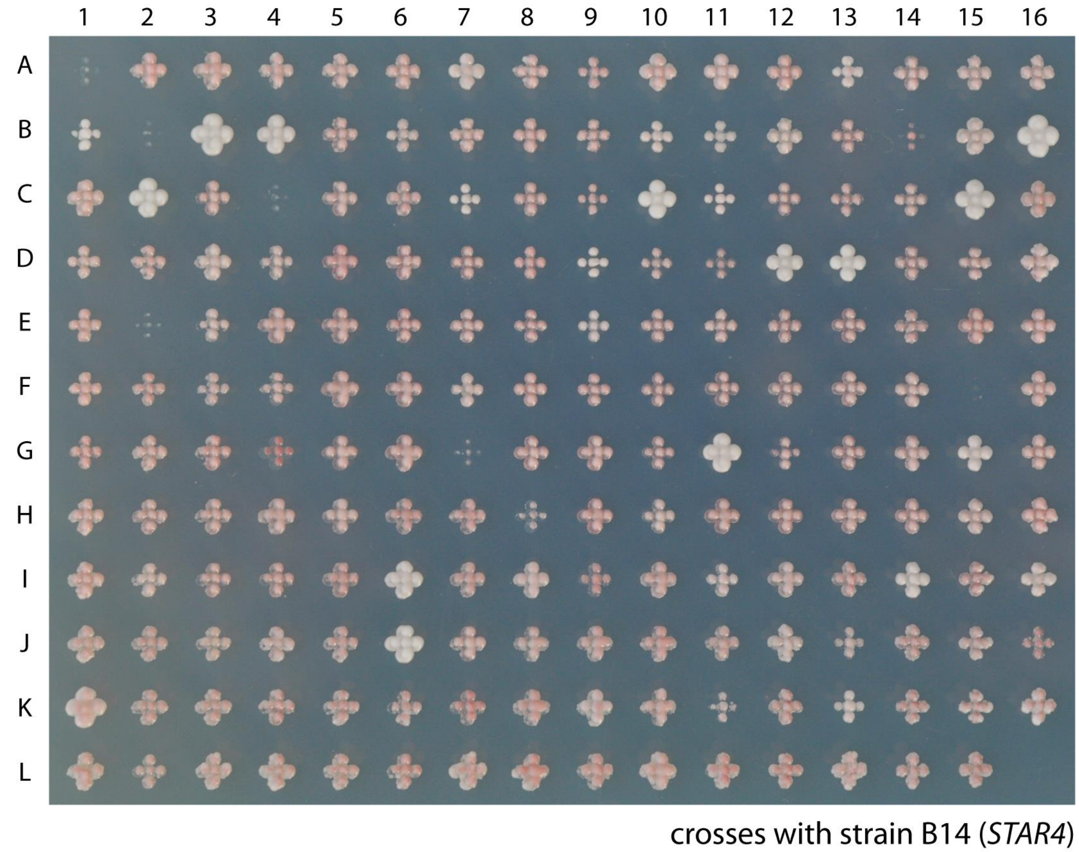

Figure S5. Second screen with *STAR4* boundary. Same as Figure S1, with strain B14 in which *IR-R*$^{+}$is replaced with the *STAR4* element.

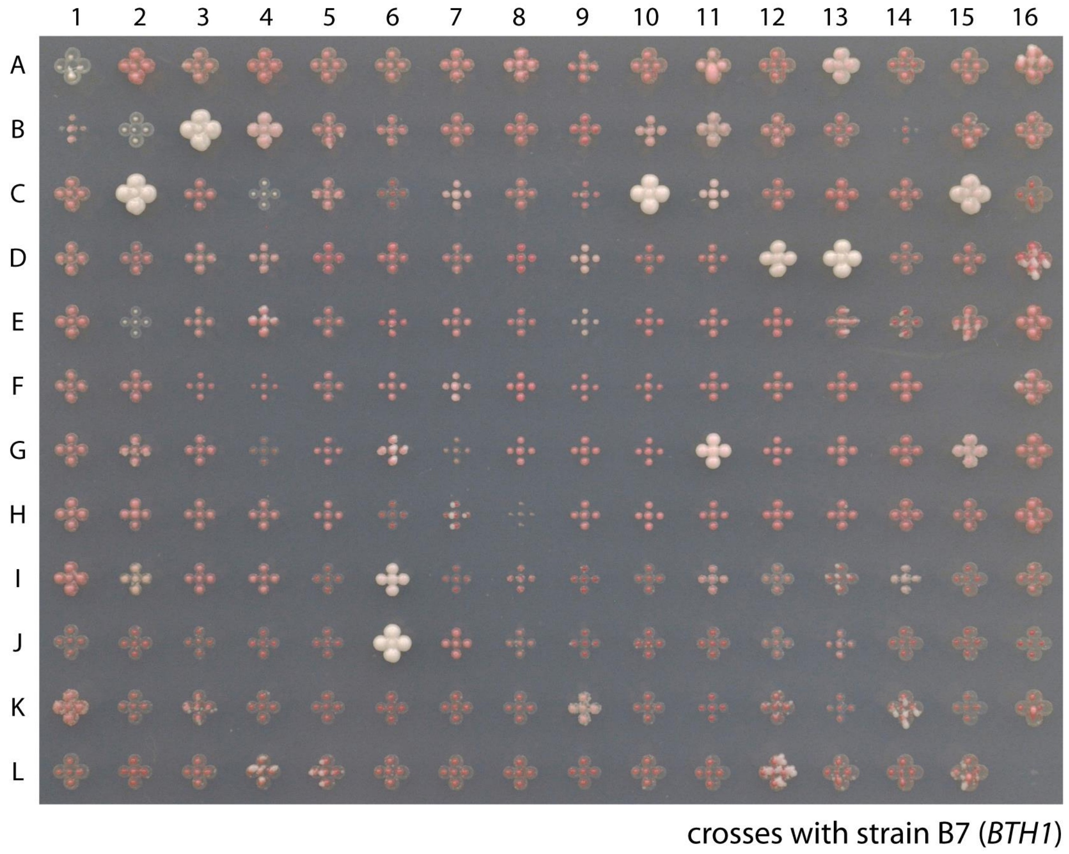

Figure S6. Second screen with *BTH1* boundary. Same as Figure S1, with strain B7 in which *IR-R*$^{+}$is replaced with the *BTH1* element.

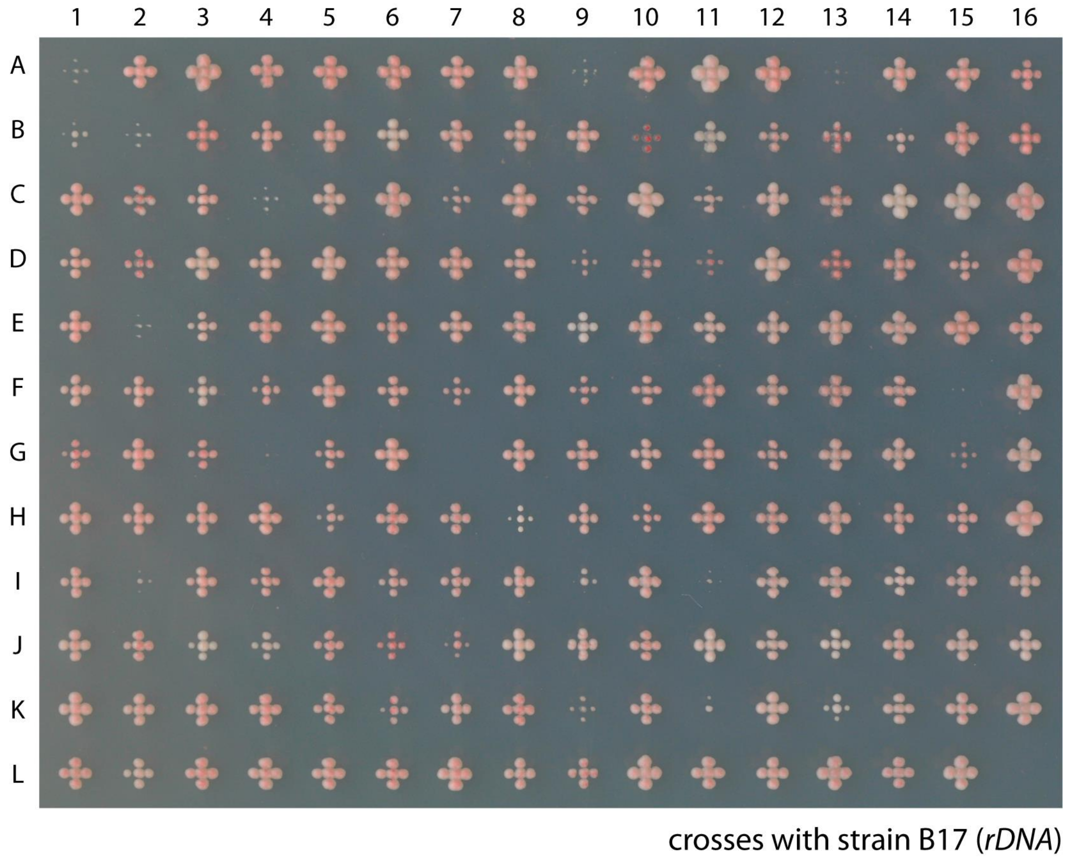

Figure S7. Second screen with *rDNA* boundary. Same as Figure S1, with strain B17 in which *IR-R*$^{+}$is replaced with an *rDNA* repeat.

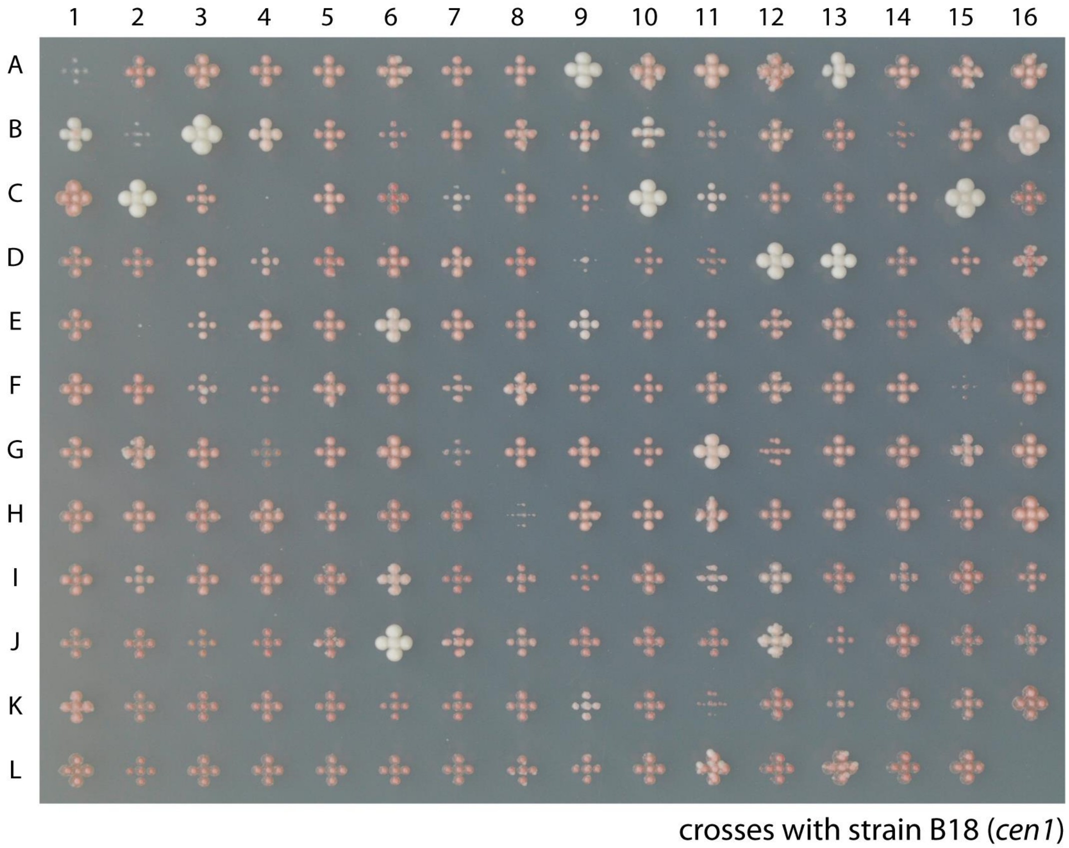

Figure S8. Second screen with *cen1* boundary. Same as Figure S1, with strain B18 in which *IR-R*$^{+}$is replaced with the *cen1* element.

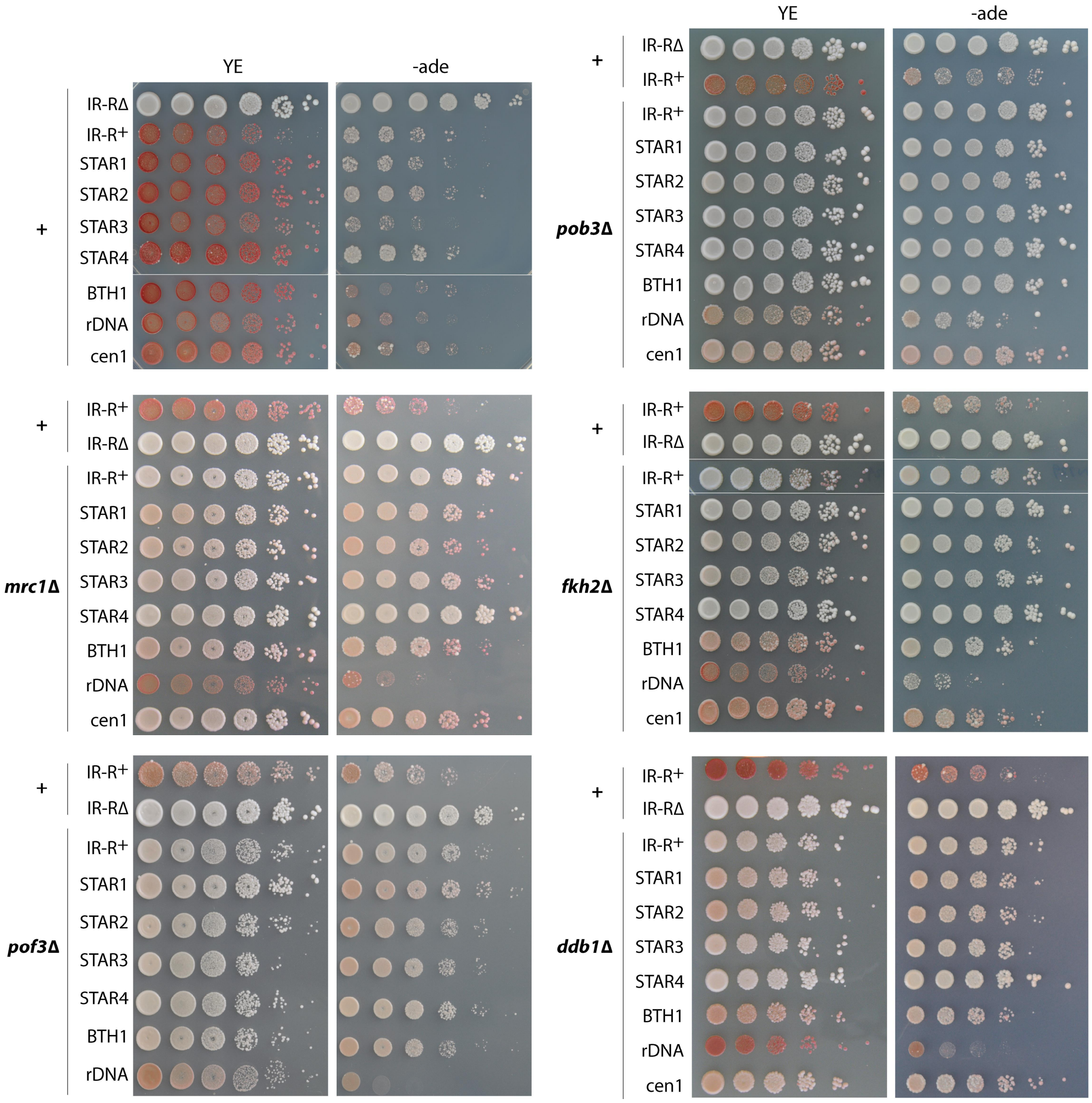

Figure S9. Third screen: effect of Pob3, Mrc1, Fkh2, Pof3, and Ddb1 on heterochromatic silencing at *(EcoRV)::ade6*$^+$. Ten-fold serial dilutions of cell suspensions were spotted on medium with a low adenine concentration (YE) or no adenine. The wild-type series ($^+$) shows PG3947 (IR-Rφ) together with the query strains used in the screen: B19, B15, B16, B6, B14, B7, B17, and B18. In the following panels, PG3950 (IR-R$^+$) and PG3947 (IR-Rφ) were spotted on all plates as standards for *(EcoRV)::ade6*$^+$repression and derepression respectively. Strains used for *pob3Δ* series: DN173, DN169, DN170, DN166, DN168, DN167, DN171, DN172; strains used for *mrc1Δ* series: LJ102, LJ117, LJ118, LJ114, LJ116, LJ115, LJ119, LJ120; strains used for *fkh2Δ* series: DN129, DN125, DN126, DN122, DN124, DN123, DN127; strains used for *pof3Δ* series: LJ220, LJ224, LJ225, LJ221, LJ223, LJ222, LJ226; strains used for *ddb1Δ* series: DN105, DN101, DN102, DN98, DN100, DN101, DN103.

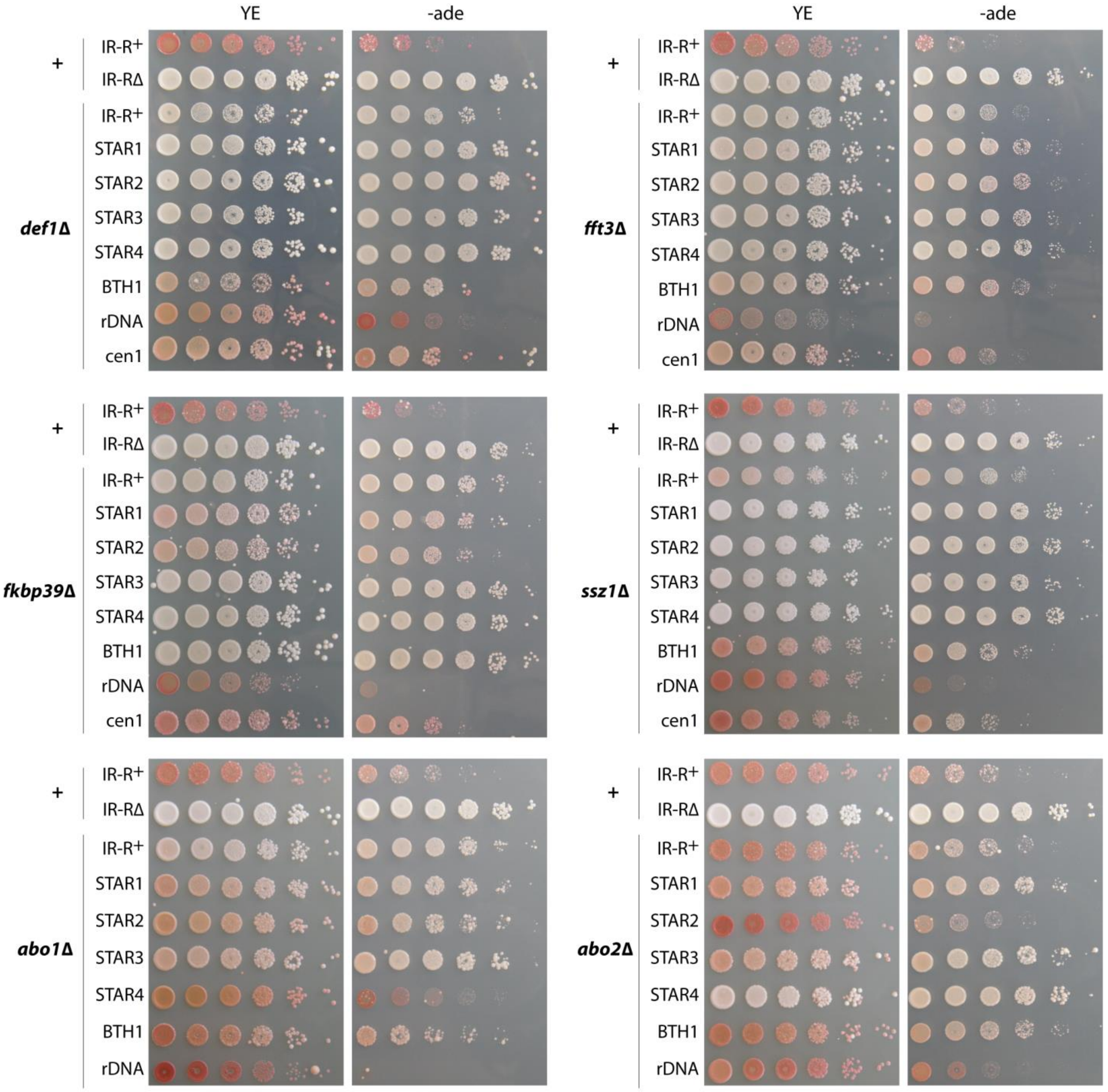

Figure S10. Third screen: effect of Def1, Fft3, Fkbp39, Ssz1, Abo1 and Abo2 on heterochromatic silencing at *(EcoRV)::ade6*$^+$. As in Figure S9, PG3950 (IR-R$^+$) and PG3947 (IR-Rφ) were spotted on all plates as standards for *(EcoRV)::ade6*$^+$ repression and derepression respectively. Strains used for *def1Δ* series: LJ100, LJ152, LJ153, LJ149, LJ151, LJ150, LJ154, LJ155; strains used for *fft3Δ* series: LJ103, LJ124, LJ125, LJ121, LJ123, LJ122, LJ126, LJ127; strains used for *fkbp39Δ* series: LJ104, LJ131, LJ132, LJ128, LJ130, LJ129, LJ133, LJ134; strains used for *ssz1Δ* series: LJ203, LJ202, LJ111, LJ107, LJ109, LJ108, LJ112, LJ113; strains used for *abo1*φ series: LJ199, DB44, DB45, DB41, DB43, DB42, DB46; strains used for *abo2Δ* series: LJ105, DB52, DB53, DB49, DB51, DB50, DB54. See Figure S9 for control strains with ectopic boundary elements in wild-type background.

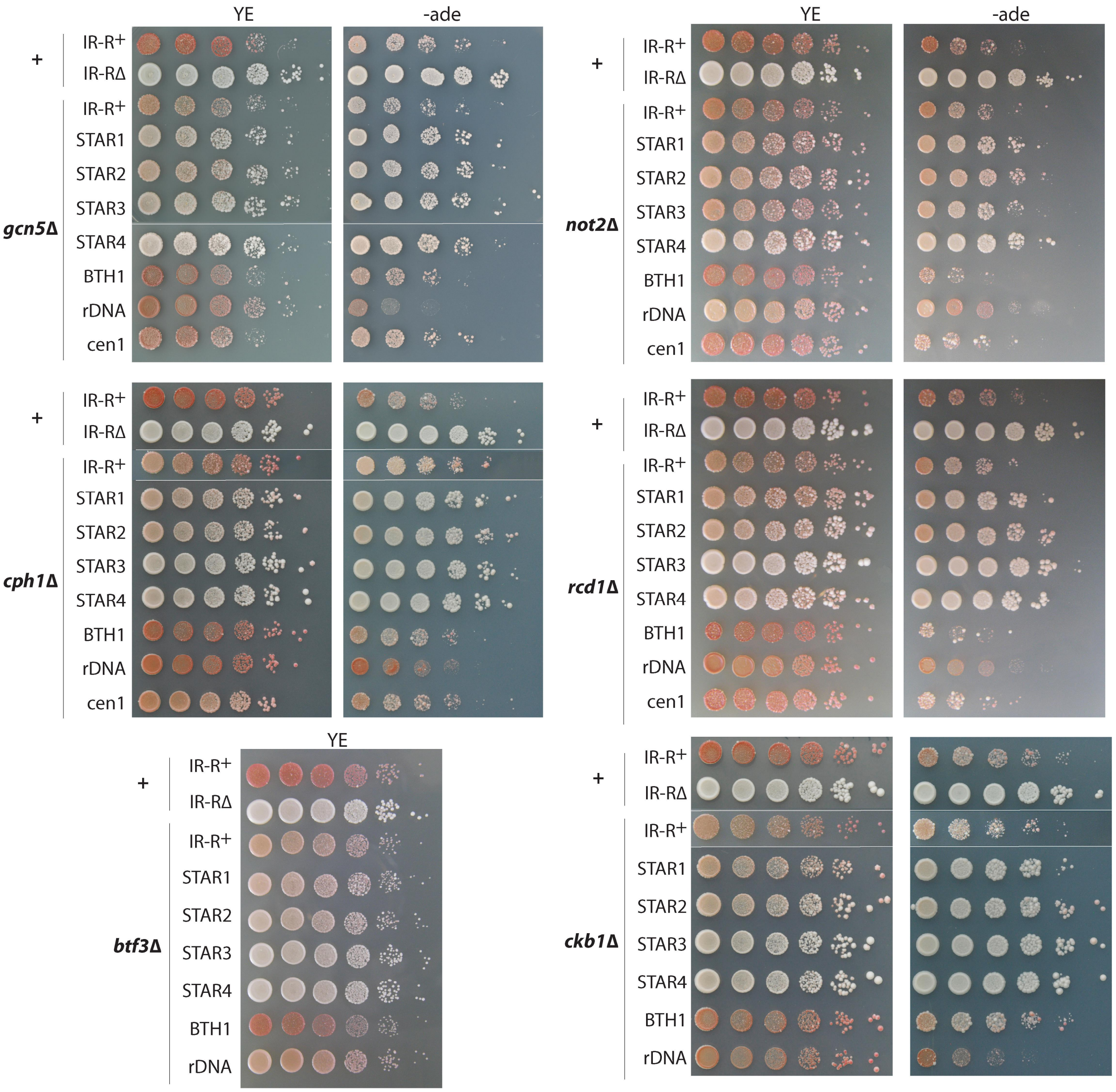

Figure S11. Third screen: effect of Gcn5, Not2, Cph1, Rcd1, Btf3, and Ckb1 on heterochromatic silencing at *(EcoRV)::ade6*$^{+}$. As in Figure S9, PG3950 (*IR-R*$^{+}$) and PG3947 (*IR-R*φ) were spotted on all plates as standards for *(EcoRV)::ade6*$^{+}$ repression and derepression respectively. Strains used for *gcn5Δ* series: PA91-PA98; strains used for *not2Δ* series: DB105, DB4, DB5, DB1, DB3, DB2, DB6, DB7; strains used for *cph1Δ* series: DN121, DN117, DN118, DN114, DN116, DN115, DN119, DN120; strains used for *rcd1Δ* series: DB112, DB60, DB61, DB57, DB59, DB58, DB62, DB63; strains used for *btf3Δ* series: DB16, DB20,DB21,DB17, DB19, DB18, DB22, DB23; strains used for *ckb1Δ* series: DN97, DN93, DN94, DN90, DN92, DN91, DN95. See Figure S9 for control strains with ectopic boundary elements in wild-type background.

Table S3. Strains and their genotypes.

Strain	Genotype
**Strains used for strain constructions and plating controls**	
PG3947	mat3-M (EcoRV)::ade6^{+} IR-RΔ (BlpI)::LEU2 leu1-32 ura4-D18 ade6-DN/N
PG3950	mat3-M (EcoRV)::ade6^{+} (BlpI)::LEU2 leu1-32 ura4-D18 ade6-DN/N
PC152	mat3-M (EcoRV)::ade6^{+} IR-RΔ (SpeI)::(STAR2) (BlpI)::LEU2 leu1-32 ura4-D18 ade6-DN/N
PT600	mat3-M (EcoRV)::ade6^{+} IR-RΔ (SpeI)::(STAR2-S) (BlpI)::LEU2 leu1-32 ura4-D18 ade6-DN/N
**Query strains used in library screens**	
B6	(XmnI)::ura4^{+} mat2-P mat3-M (EcoRV)::ade6^{+} IR-RΔ (SpeI)::(BTH1) (BlpI)::LEU2 leu1-32 ura4-D18 ade6-DN/N arg12Δ::natR
B7	(XmnI)::ura4^{+} mat2-P mat3-M (EcoRV)::ade6^{+} IR-RΔ (SpeI)::(STAR3) (BlpI)::LEU2 leu1-32 ura4-D18 ade6-DN/N arg12Δ::natR
B14	(XmnI)::ura4^{+} mat2-P mat3-M (EcoRV)::ade6^{+} IR-RΔ (SpeI)::(STAR4) (BlpI)::LEU2 leu1-32 ura4-D18 ade6-DN/N arg12Δ::natR
B15	(XmnI)::ura4^{+} mat2-P mat3-M (EcoRV)::ade6^{+} IR-RΔ (SpeI)::(STAR1) (BlpI)::LEU2 leu1-32 ura4-D18 ade6-DN/N arg12Δ::natR
B16	(XmnI)::ura4^{+} mat2-P mat3-M (EcoRV)::ade6^{+} IR-RΔ (SpeI)::(STAR2) (BlpI)::LEU2 leu1-32 ura4-D18 ade6-DN/N arg12Δ::natR
B17	(XmnI)::ura4^{+} mat2-P mat3-M (EcoRV)::ade6^{+} R-RΔ (SpeI)::(rDNA) (BlpI)::LEU2 leu1-32 ura4-D18 ade6-DN/N arg12Δ::natR
B18	(XmnI)::ura4^{+} mat2-P mat3-M (EcoRV)::ade6^{+} IR-RΔ (SpeI)::(cen1) (BlpI)::LEU2 leu1-32 ura4-D18 ade6-DN/N arg12Δ::natR
B19	(XmnI)::ura4^{+} mat2-P mat3-M (EcoRV)::ade6^{+} leu1-32 ura4-D18 ade6-DN/N arg12Δ::natR
**Strains in Figure 4**	
LJ99	mat3-M (EcoRV)::ade6^{+} (BlpI)::LEU2 clr4Δ::kanR leu1-32 ura4-D18 ade6-DN/N
PG3950	mat3-M (EcoRV)::ade6^{+} (BlpI)::LEU2 leu1-32 ura4-D18 ade6-DN/N
LJ102	mat3-M (EcoRV)::ade6^{+} (BlpI)::LEU2 mrc1Δ::kanR leu1-32 ura4-D18 ade6-DN/N
LJ100	mat3-M (EcoRV)::ade6^{+} (BlpI)::LEU2 def1Δ::kanR leu1-32 ura4-D18 ade6-DN/N
LJ220	mat3-M (EcoRV)::ade6^{+} (BlpI)::LEU2 pof3Δ::kanR leu1-32 ura4-D18 ade6-DN/N
LJ203	mat3-M (EcoRV)::ade6^{+} (BlpI)::LEU2 ssz1Δ::kanR leu1-32 ura4-D18 ade6-DN/N
LJ104	mat3-M (EcoRV)::ade6^{+} (BlpI)::LEU2 fkbp39Δ::kanR leu1-32 ura4-D18 ade6-DN/N
LJ103	mat3-M (EcoRV)::ade6^{+} (BlpI)::LEU2 fft3Δ::kanR leu1-32 ura4-D18 ade6-DN/N
LJ199	mat3-M (EcoRV)::ade6^{+} (BlpI)::LEU2 abo1Δ::kanR leu1-32 ura4-D18 ade6-DN/N
LJ105	mat3-M (EcoRV)::ade6^{+} (BlpI)::LEU2 abo2Δ::kanR leu1-32 ura4-D18 ade6-DN/N
PM20	mat3-M(EcoRV)::ade6^{+}swi1::kanR leu1^{+}CO3-CFP ura4-D18 ade6-DN/N his7^{+}::lacI-GFP his2[::hph1-ura4^{+}-lacOp]
PM24	mat3-M(EcoRV)::ade6^{+} swi3::kanR leu1^{+}CO3-CFP ura4-D18 ade6-DN/N his7^{+}::lacI-GFP his2[::hph1-ura4^{+}-lacOp]
**Strains in Figure 6**	
LJ177	mat3-M (EcoRV)::ura4^{+} clr4Δ::kanR leu1-32 ura4-DS/E ade6-210
PG1899	mat3-M (EcoRV)::ura4^{+} leu1-32 ura4-DS/E ade6-216
LJ176	mat3-M (EcoRV)::ura4^{+} mrc1Δ::kanR leu1-32 ura4-DS/E ade6-210
LJ178	mat3-M (EcoRV)::ura4^{+} pof3Δ::kanR leu1-32 ura4-DS/E ade6-210
LJ179	mat3-M (EcoRV)::ura4^{+} def1Δ::kanR leu1-32 ura4-DS/E ade6-210

LJ175	mat3-M (EcoRV)::ura4^{+} ssz1Δ::kanR leu1-32 ura4-DS/E ade6-210
LJ196	mat3-M (EcoRV)::ura4^{+} fkbp39Δ::kanR leu1-32 ura4-DS/E ade6-210
LJ197	mat3-M (EcoRV)::ura4^{+} fft3Δ::kanR leu1-32 ura4-DS/E ade6-210
**Strains in Figure 7**	
PA71	mat3-M (EcoRV)::ade6^{+} (BlpI)::LEU2 mcl1Δ::kanR leu1-32 ura4-D18 ade6-DN/N
PA72	mat3-M (EcoRV)::ade6^{+} IR-RΔ (SpeI)::(STAR2) (BlpI)::LEU2 mcl1Δ::kanR leu1-32 ura4-D18 ade6-DN/N
PA73	mat3-M (EcoRV)::ade6^{+} IR-RΔ (SpeI)::(STAR2-S) (BlpI)::LEU2 mcl1Δ::kanR leu1-32 ura4-D18 ade6-DN/N
PA74	mat3-M (EcoRV)::ade6^{+} IR-RΔ (BlpI)::LEU2 mcl1Δ::kanR leu1-32 ura4-D18 ade6-DN/N
PA82	mat3-M (EcoRV)::ade6^{+} (BlpI)::LEU2 wpl1Δ::kanR leu1-32 ura4-D18 ade6-DN/N
PA83	mat3-M (EcoRV)::ade6^{+} IR-RΔ (SpeI)::(STAR2) (BlpI)::LEU2 wpl1Δ::kanR leu1-32 ura4-D18 ade6-DN/N
PA84	mat3-M (EcoRV)::ade6^{+} IR-RΔ (SpeI)::(STAR2-S) (BlpI)::LEU2 wpl1Δ::kanR leu1-32 ura4-D18 ade6-DN/N
PA85	mat3-M (EcoRV)::ade6^{+} IR-RΔ (BlpI)::LEU2 l wpl1Δ::kanR leu1-32 ura4-D18 ade6-DN/N
**Strains in Figure 8**	
LJ171	h^{A} clr4Δ::kanR leu1-32 ura4-DS/E ade6-210/216 Chr16 m23:ura4^{+}-TEL(72)
FY520	h^{+}leu1-32 ura4-DS/E ade6-210/216 Chr16 m23:ura4-TEL(72)
LJ163	h^{A} mrc1Δ::kanR leu1-32 ura4-DS/E ade6-210/216 Chr16 m23:ura4^{+}-TEL(72)
LJ172	h^{A} pof3Δ::kanR leu1-32 ura4-DS/E ade6-210/216 Chr16 m23:ura4^{+}-TEL(72)
LJ173	h^{A} def1Δ::kanR leu1-32 ura4-DS/E ade6-210/216 Chr16 m23:ura4^{+}-TEL(72)
LJ162	h^{A} ssz1Δ::kanR leu1-32 ura4-DS/E ade6-210/216 Chr16 m23:ura4^{+}-TEL(72)
LJ174	h^{A} fkbp39Δ::kanR leu1-32 ura4-DS/E ade6-210/216 Chr16 m23:ura4^{+}-TEL(72)
LJ165	h^{A} fft3Δ::kanR leu1-32 ura4-DS/E ade6-210/216 Chr16 m23:ura4^{+}-TEL(72)
LJ170	^{A}h btf3Δ::kanR leu1-32 ura4-DS/E ade6-210/216 Chr16 m23:ura4^{+}-TEL(72)
LJ168	h^{A} rif1Δ::kanR leu1-32 ura4-DS/E ade6-210/216 Chr16 m23:ura4^{+}-TEL(72)
LJ161	h^{A} abo1Δ::kanR leu1-32 ura4-DS/E ade6-210/216 Chr16 m23:ura4^{+}-TEL(72)
LJ166	h^{A} abo2Δ::kanR leu1-32 ura4-DS/E ade6-210/216 Chr16 m23:ura4-TEL(72)
LJ185	h^{A} clr4Δ::kanR leu1-32 ura4-DS/E ade6-210 otr1(SphI)::ura4^{+}
FY648	h^{+}leu1-32 ura4-DS/E ade6-210 otr1(SphI)::ura4^{+}
LJ180	h^{A} mrc1Δ::kanR leu1-32 ura4-DS/E ade6-210 otr1(SphI)::ura4^{+}
LJ186	h^{A} pof3Δ::kanR leu1-32 ura4-DS/E ade6-210 otr1(SphI)::ura4^{+}
LJ190	h^{A} def1Δ::kanR leu1-32 ura4-DS/E ade6-210 otr1(SphI)::ura4^{+}
LJ192	h^{A} ssz1Δ::kanR leu1-32 ura4-DS/E ade6-210 otr1(SphI)::ura4^{+}
LJ187	h^{A} fkbp39Δ::kanR leu1-32 ura4-DS/E ade6-210 otr1(SphI)::ura4^{+}
LJ183	h^{A} btf3Δ::kanR leu1-32 ura4-DS/Eade6-210 otr1(SphI)::ura4^{+}
LJ181	h^{A} rif1Δ::kanR leu1-32 ura4-DS/E ade6-210 otr1(SphI)::ura4^{+}
LJ191	h^{A} abo1Δ::kanR leu1-32 ura4-DS/E ade6-210 otr1(SphI)::ura4^{+}
LJ189	h^{A} abo2Δ::kanR leu1-32 ura4-DS/E ade6-210 otr1(SphI)::ura4^{+}

**Strains in Figure S9**	
***mrc1Δ* series**	
LJ102	*mat3-M (EcoRV)::ade6*$^{+}$ *(BlpI)::LEU2 mrc1Δ::kanR leu1-32 ura4-D18 ade6-DN/N*
LJ117	*(XmnI)::ura4*$^{+}$ *mat2-P mat3-M (EcoRV)::ade6*$^{+}$ *IR-RΔ (SpeI)::(STAR1) (BlpI)::LEU2 mrc1Δ::kanR leu1-32 ura4-D18 ade6-DN/N arg12Δ::natR*
LJ118	*(XmnI)::ura4*$^{+}$ *mat2-P mat3-M (EcoRV)::ade6*$^{+}$ *IR-RΔ (SpeI)::(STAR2) (BlpI)::LEU2 mrc1Δ::kanR leu1-32 ura4-D18 ade6-DN/N arg12Δ::natR*
LJ114	*(XmnI)::ura4*$^{+}$ *mat2-P mat3-M (EcoRV)::ade6*$^{+}$ *IR-RΔ (SpeI)::(STAR3) (BlpI)::LEU2 mrc1Δ::kanR leu1-32 ura4-D18 ade6-DN/N arg12Δ::natR*
LJ116	*mat2-M (XmnI)::ura4* $^{+}$ *mat3-M (EcoRV)::ade6*$^{+}$ *IR-RΔ (SpeI)::(STAR4) (BlpI)::LEU2 mrc1Δ::kanR leu1-32 ura4-D18 ade6-DN/N arg12Δ::natR*
LJ115	*mat2-M (XmnI)::ura4* $^{+}$ *mat3-M (EcoRV)::ade6*$^{+}$ *IR-RΔ (SpeI)::(BTH1) (BlpI)::LEU2 mrc1Δ::kanR leu1-32 ura4-D18 ade6-DN/N arg12Δ::natR*
LJ119	*(XmnI)::ura4*$^{+}$ *mat2-P mat3-M (EcoRV)::ade6*$^{+}$ *IR-RΔ (SpeI)::(rDNA) (BlpI)::LEU2 mrc1Δ::kanR leu1-32 ura4-D18 ade6-DN/N arg12Δ::natR*
LJ120	*(XmnI)::ura4*$^{+}$ *mat2-P mat3-M (EcoRV)::ade6*$^{+}$ *IR-RΔ (SpeI)::(edge cen1) (BlpI)::LEU2 mrc1Δ::kanR leu1-32 ura4-D18 ade6-DN/N arg12Δ::natR*
***pof3Δ* series**	
LJ220	*mat3-M (EcoRV)::ade6*$^{+}$ *(BlpI)::LEU2 pof3Δ::kanR leu1-32 ura4-D18 ade6-DN/N*
LJ224	*mat3-M (EcoRV)::ade6*$^{+}$ *IR-RΔ (SpeI)::(STAR1) (BlpI)::LEU2 pof3Δ::kanR leu1-32 ura4-D18 ade6-DN/N*
LJ225	*mat3-M (EcoRV)::ade6*$^{+}$ *IR-RΔ (SpeI)::(STAR2) (BlpI)::LEU2 pof3Δ::kanR leu1-32 ura4-D18 ade6-DN/N*
LJ221	*mat3-M (EcoRV)::ade6*$^{+}$ *IR-RΔ (SpeI)::(STAR3) (BlpI)::LEU2 pof3Δ::kanR leu1-32 ura4-D18 ade6-DN/N*
LJ223	*mat3-M (EcoRV)::ade6*$^{+}$ *IR-RΔ (SpeI)::(STAR4) (BlpI)::LEU2 pof3Δ::kanR leu1-32 ura4-D18 ade6-DN/N*
LJ222	*mat3-M (EcoRV)::ade6*$^{+}$ *IR-RΔ (SpeI)::(BTH1) (BlpI)::LEU2 pof3Δ::kanR leu1-32 ura4-D18 ade6-DN/N*
LJ226	*mat3-M (EcoRV)::ade6*$^{+}$ *IR-RΔ (SpeI)::(rDNA) (BlpI)::LEU2 pof3Δ::kanR leu1-32 ura4-D18 ade6-DN/N*
pob3Δ series	
DN173	*mat3-M (EcoRV)::ade6*$^{+}$ *(BlpI)::LEU2 pob3Δ::kanR leu1-32 ura4-D18 or DS/E ade6-DN/N*
DN169	*(XmnI)::ura4*$^{+}$ *mat2-P mat3-M (EcoRV)::ade6*$^{+}$ *IR-RΔ (SpeI)::(STAR1) (BlpI)::LEU2 pob3Δ::kanR leu1-32 ura4-D18 or DS/E ade6-DN/N*
DN170	*(XmnI)::ura4*$^{+}$ *mat2-P mat3-M (EcoRV)::ade6*$^{+}$ *IR-RΔ (SpeI)::(STAR2) (BlpI)::LEU2 pob3Δ::kanR leu1-32 ura4-D18 or DS/E ade6-DN/N*
DN166	*(XmnI)::ura4*$^{+}$ *mat2-P mat3-M (EcoRV)::ade6*$^{+}$ *IR-RΔ (SpeI)::(STAR3) (BlpI)::LEU2 pob3Δ::kanR leu1-32 ura4-D18 or DS/E ade6-DN/N*
DN168	*(XmnI)::ura4*$^{+}$ *mat2-P mat3-M (EcoRV)::ade6*$^{+}$ *IR-RΔ (SpeI)::(STAR4) (BlpI)::LEU2 pob3Δ::kanR leu1-32 ura4-D18 or DS/E ade6-DN/N*
DN167	*(XmnI)::ura4*$^{+}$ *mat2-P mat3-M (EcoRV)::ade6*$^{+}$ *IR-RΔ (SpeI)::(BTH1) (BlpI)::LEU2 pob3Δ::kanR leu1-32 ura4-D18 or DS/E ade6-DN/N*
DN171	*mat2-M (XmnI)::ura4* $^{+}$ *mat3-M (EcoRV)::ade6*$^{+}$ *IR-RΔ (SpeI)::(rDNA) (BlpI)::LEU2 pob3Δ::kanR leu1-32 ura4-D18 or DS/E ade6-DN/N*
DN172	*(XmnI)::ura4*$^{+}$ *mat2-P mat3-M (EcoRV)::ade6*$^{+}$ *IR-RΔ (SpeI)::(cen1 NheI) (BlpI)::LEU2 pob3Δ::kanR leu1-32 ura4-D18 or DS/E ade6-DN/N*

fkh2Δ series	
DN129	*mat3-M (EcoRV)::ade6⁺ (BlpI)::LEU2 fkh2Δ::kanR leu1-32 ura4-D18 or DS/E ade6-DN/N*
DN125	*(XmnI)::ura4⁺ mat2-P mat3-M (EcoRV)::ade6⁺ IR-RΔ (SpeI)::(STAR1) (BlpI)::LEU2 fkh2Δ::kanR leu1-32 ura4-D18 or DS/E ade6-DN/N*
DN126	*(XmnI)::ura4⁺ mat2-P mat3-M (EcoRV)::ade6⁺ IR-RΔ (SpeI)::(STAR2) (BlpI)::LEU2 fkh2Δ::kanR leu1-32 ura4-D18 or DS/E ade6-DN/N*
DN122	*(XmnI)::ura4⁺ mat2-P mat3-M (EcoRV)::ade6⁺ IR-RΔ (SpeI)::(STAR3) (BlpI)::LEU2 fkh2Δ::kanR leu1-32 ura4-D18 or DS/E ade6-DN/N*
DN124	*(XmnI)::ura4⁺ mat2-P mat3-M (EcoRV)::ade6⁺ IR-RΔ (SpeI)::(STAR4) (BlpI)::LEU2 fkh2Δ::kanR leu1-32 ura4-D18 or DS/E ade6-DN/N*
DN123	*(XmnI)::ura4⁺ mat2-P mat3-M (EcoRV)::ade6⁺ IR-RΔ (SpeI)::(BTH1) (BlpI)::LEU2 fkh2Δ::kanR leu1-32 ura4-D18 or DS/E ade6-DN/N*
DN127	*(XmnI)::ura4⁺ mat2-P mat3-M (EcoRV)::ade6⁺ IR-RΔ (SpeI)::(rDNA) (BlpI)::LEU2 fkh2Δ::kanR leu1-32 ura4-D18 or DS/E ade6-DN/N*
ddb1Δ series	
DN105	*mat3-M (EcoRV)::ade6⁺ (BlpI)::LEU2 ddb1Δ::kanR leu1-32 ura4-D18 or DS/E ade6-DN/N*
DN101	*(XmnI)::ura4⁺ mat2-P mat3-M (EcoRV)::ade6⁺ IR-RΔ (SpeI)::(STAR1) (BlpI)::LEU2 ddb1Δ::kanR leu1-32 ura4-D18 or DS/E ade6-DN/N*
DN102	*(XmnI)::ura4⁺ mat2-P mat3-M (EcoRV)::ade6⁺ IR-RΔ (SpeI)::(STAR2) (BlpI)::LEU2 ddb1Δ::kanR leu1-32 ura4-D18 or DS/E ade6-DN/N*
DN98	*(XmnI)::ura4⁺ mat2-P mat3-M (EcoRV)::ade6⁺ IR-RΔ (SpeI)::(STAR3) (BlpI)::LEU2 ddb1Δ::kanR leu1-32 ura4-D18 or DS/E ade6-DN/N*
DN100	*(XmnI)::ura4⁺ mat2-P mat3-M (EcoRV)::ade6⁺ IR-RΔ (SpeI)::(STAR4) (BlpI)::LEU2 ddb1Δ::kanR leu1-32 ura4-D18 or DS/E ade6-DN/N*
DN101	*(XmnI)::ura4⁺ mat2-P mat3-M (EcoRV)::ade6⁺ IR-RΔ (SpeI)::(BTH1) (BlpI)::LEU2 ddb1Δ::kanR leu1-32 ura4-D18 or DS/E ade6-DN/N*
DN103	*(XmnI)::ura4⁺ mat2-P mat3-M (EcoRV)::ade6⁺ IR-RΔ (SpeI)::(rDNA) (BlpI)::LEU2 ddb1Δ::kanR leu1-32 ura4-D18 or DS/E ade6-DN/N*
**Strains in Figure S10**	
def1Δ **series**	
LJ100	*mat3-M (EcoRV)::ade6⁺ (BlpI)::LEU2 def1Δ::kanR leu1-32 ura4-D18 ade6-DN/N*
LJ152	*(XmnI)::ura4⁺ mat2-P mat3-M (EcoRV)::ade6⁺ IR-RΔ (SpeI)::(STAR1) (BlpI)::LEU2 def1Δ::kanR leu1-32 ura4-D18 ade6-DN/N arg12Δ::natR*
LJ153	*(XmnI)::ura4⁺ mat2-P mat3-M (EcoRV)::ade6⁺ IR-RΔ (SpeI)::(STAR2) (BlpI)::LEU2 def1Δ::kanR leu1-32 ura4-D18 ade6-DN/N arg12Δ::natR*
LJ149	*(XmnI)::ura4⁺ mat2-P mat3-M (EcoRV)::ade6⁺ IR-RΔ (SpeI)::(STAR3) (BlpI)::LEU2 def1Δ::kanR leu1-32 ura4-D18 ade6-DN/N arg12Δ::natR*
LJ151	*(XmnI)::ura4⁺ mat2-P mat3-M (EcoRV)::ade6⁺ IR-RΔ (SpeI)::(STAR4) (BlpI)::LEU2 def1Δ::kanR leu1-32 ura4-D18 ade6-DN/N arg12Δ::natR*
LJ150	*(XmnI)::ura4⁺ mat2-P mat3-M (EcoRV)::ade6⁺ IR-RΔ (SpeI)::(BTH1) (BlpI)::LEU2 def1Δ::kanR leu1-32 ura4-D18 ade6-DN/N arg12Δ::natR*
LJ154	*(XmnI)::ura4⁺ mat2-P mat3-M (EcoRV)::ade6⁺ IR-RΔ (SpeI)::(rDNA) (BlpI)::LEU2 def1Δ::kanR leu1-32 ura4-D18 ade6-DN/N arg12Δ::natR*
LJ155	*(XmnI)::ura4⁺ mat2-P mat3-M (EcoRV)::ade6⁺ IR-RΔ (SpeI)::(edge cen1) (BlpI)::LEU2 def1Δ::kanR leu1-32 ura4-D18 ade6-DN/N arg12Δ::natR*

fft3Δ **series**	
LJ103	*mat3-M (EcoRV)::*$ade6^+$ *(BlpI)::LEU2 fft3Δ::kanR leu1-32 ura4-D18 ade6-DN/N*
LJ124	*(XmnI)::*$ura4^+$ *mat2-P mat3-M (EcoRV)::*$ade6^+$ *IR-RΔ (SpeI)::(STAR1) (BlpI)::LEU2 fft3Δ::kanR leu1-32 ura4-D18 ade6-DN/N arg12Δ::natR*
LJ125	*(XmnI)::*$ura4^+$ *mat2-P mat3-M (EcoRV)::*$ade6^+$ *IR-RΔ (SpeI)::(STAR2) (BlpI)::LEU2 fft3Δ::kanR leu1-32 ura4-D18 ade6-DN/N arg12Δ::natR*
LJ121	*(XmnI)::*$ura4^+$ *mat2-P mat3-M (EcoRV)::*$ade6^+$ *IR-RΔ (SpeI)::(STAR3) (BlpI)::LEU2 fft3Δ::kanR leu1-32 ura4-D18 ade6-DN/N arg12Δ::natR*
LJ123	*(XmnI)::*$ura4^+$ *mat2-P mat3-M (EcoRV)::*$ade6^+$ *IR-RΔ (SpeI)::(STAR4) (BlpI)::LEU2 fft3Δ::kanR leu1-32 ura4-D18 ade6-DN/N arg12Δ::natR*
LJ122	*(XmnI)::*$ura4^+$ *mat2-P mat3-M (EcoRV)::*$ade6^+$ *IR-RΔ (SpeI)::(BTH1) (BlpI)::LEU2 fft3Δ::kanR ura4-leu1-32 D18 ade6-DN/N arg12Δ::natR*
LJ126	*(XmnI)::*$ura4^+$ *mat2-P mat3-M (EcoRV)::*$ade6^+$ *IR-RΔ (SpeI)::(rDNA) (BlpI)::LEU2 fft3Δ::kanR leu1-32 ura4-D18 ade6-DN/N arg12Δ::natR*
LJ127	*(XmnI)::*$ura4^+$ *mat2-P mat3-M (EcoRV)::*$ade6^+$ *IR-RΔ (SpeI)::(edge of cen1 RHS NheI) (BlpI)::LEU2 fft3Δ::kanR leu1-32 ura4-D18 ade6-DN/N arg12Δ::natR*
fkbp39Δ **series**	
LJ104	*mat3-M (EcoRV)::*$ade6^+$ *(BlpI)::LEU2 fkbp39Δ::kanR leu1-32 ura4-D18 ade6-DN/N*
LJ131	*(XmnI)::*$ura4^+$ *mat2-P mat3-M (EcoRV)::*$ade6^+$ *IR-RΔ (SpeI)::(STAR1) (BlpI)::LEU2 fkbp39Δ::kanR leu1-32 ura4-D18 ade6-DN/N arg12Δ::natR*
LJ132	*(XmnI)::*$ura4^+$ *mat2-P mat3-M (EcoRV)::*$ade6^+$ *IR-RΔ (SpeI)::(STAR2) (BlpI)::LEU2 fkbp39Δ::kanR leu1-32 ura4-D18 ade6-DN/N arg12Δ::natR*
LJ128	*(XmnI)::*$ura4^+$ *mat2-P mat3-M (EcoRV)::*$ade6^+$ *IR-RΔ (SpeI)::(STAR3) (BlpI)::LEU2 fkbp39Δ::kanR leu1-32 ura4-D18 ade6-DN/N arg12Δ::natR*
LJ130	*(XmnI)::*$ura4^+$ *mat2-P mat3-M (EcoRV)::*$ade6^+$ *IR-RΔ (SpeI)::(STAR4) (BlpI)::LEU2 fkbp39Δ::kanR ura4-D18 ade6-DN/N arg12Δ::natR*
LJ129	*(XmnI)::*$ura4^+$ *mat2-P mat3-M (EcoRV)::*$ade6^+$ *IR-RΔ (SpeI)::(BTH1) (BlpI)::LEU2 fkbp39Δ::kanR leu1-32 ura4-D18 ade6-DN/N arg12Δ::natR*
LJ133	*(XmnI)::*$ura4^+$ *mat2-P mat3-M (EcoRV)::*$ade6^+$ *IR-RΔ (SpeI)::(rDNA) (BlpI)::LEU2 fkbp39Δ::kanR leu1-32 ura4-D18 ade6-DN/N arg12Δ::natR*
LJ134	*(XmnI)::*$ura4^+$ *mat2-P mat3-M (EcoRV)::*$ade6^+$ *IR-RΔ (SpeI)::(edge cen1) (BlpI)::LEU2 fkbp39Δ::kanR leu1-32 ura4-D18 ade6-DN/N arg12Δ::natR*
ssz1Δ **series**	
LJ203	*mat3-M (EcoRV)::*$ade6^+$ *(BlpI)::LEU2 ssz1Δ::kanR leu1-32 ura4-D18 ade6-DN/N*
LJ202	*(XmnI)::*$ura4^+$ *mat2-P mat3-M (EcoRV)::*$ade6^+$ *IR-RΔ (SpeI)::(STAR1) (BlpI)::LEU2 ssz1Δ::kanR leu1-32 ura4-D18 ade6-DN/N arg12Δ::natR?*
LJ111	*(XmnI)::*$ura4^+$ *mat2-P mat3-M (EcoRV)::*$ade6^+$ *IR-RΔ (SpeI)::(STAR2) (BlpI)::LEU2 ssz1Δ::kanR leu1-32 ura4-D18 ade6-DN/N arg12Δ::natR*
LJ107	*(XmnI)::*$ura4^+$ *mat2-P mat3-M (EcoRV)::*$ade6^+$ *IR-RΔ (SpeI)::(STAR3) (BlpI)::LEU2 ssz1Δ::kanR leu1-32 ura4-D18 ade6-DN/N arg12Δ::natR*
LJ109	*(XmnI)::*$ura4^+$ *mat2-P mat3-M (EcoRV)::*$ade6^+$ *IR-RΔ (SpeI)::(STAR4) (BlpI)::LEU2 ssz1Δ::kanR leu1-32 ura4-D18 ade6-DN/N arg12Δ::natR*
LJ108	*(XmnI)::*$ura4^+$ *mat2-P mat3-M (EcoRV)::*$ade6^+$ *IR-RΔ (SpeI)::(BTH1) (BlpI)::LEU2 ssz1Δ::kanR leu1-32 ura4-D18 ade6-DN/N arg12Δ::natR*
LJ112	*(XmnI)::*$ura4^+$ *mat2-P mat3-M (EcoRV)::*$ade6^+$ *IR-RΔ (SpeI)::(rDNA) (BlpI)::LEU2 ssz1Δ::kanR leu1-32 ura4-D18 ade6-DN/N arg12Δ::natR*
LJ113	*(XmnI)::*$ura4^+$ *mat2-P mat3-M (EcoRV)::*$ade6^+$ *IR-RΔ (SpeI)::(edge cen1) (BlpI)::LEU2 ssz1Δ::kanR leu1-32 ura4-D18 ade6-DN/N arg12Δ::natR*

***abo1Δ* series**	
LJ199	*mat3-M (EcoRV)::ade6*$^{+}$ *(BlpI)::LEU2 abo1Δ::kanR leu1-32 ura4-D18 ade6-DN/N*
DB44	*(XmnI)::ura4*$^{+}$ *mat2-P mat3-M (EcoRV)::ade6 IR-RΔ (SpeI)::(STAR1) (BlpI)::LEU2 leu1-32 ura4-D18 ade6-DN/N abo1Δ::kanR*
DB45	*(XmnI)::ura4*$^{+}$ *mat2-P mat3-M (EcoRV)::ade6 IR-RΔ (SpeI)::(STAR2) (BlpI)::LEU2 leu1-32 ura4-D18 ade6-DN/N abo1Δ::kanR*
DB41	*(XmnI)::ura4*$^{+}$ *mat2-P mat3-M (EcoRV)::ade6 IR-RΔ (SpeI)::(STAR3) (BlpI)::LEU2 leu1-32 ura4-D18 ade6-DN/N abo1Δ::kanR*
DB43	*(XmnI)::ura4*$^{+}$ *mat2-P mat3-M (EcoRV)::ade6 IR-RΔ (SpeI)::(STAR4) (BlpI)::LEU2 leu1-32 ura4-D18 ade6-DN/N abo1Δ::kanR*
DB42	*(XmnI)::ura4*$^{+}$ *mat2-P mat3-M (EcoRV)::ade6 IR-RΔ (SpeI):(BTH1) (BlpI)::LEU2 leu1-32 ura4-D18 ade6-DN/N abo1Δ::kanR*
DB46	*(XmnI)::ura4*$^{+}$ *mat2-P mat3-M (EcoRV)::ade6 IR-RΔ (SpeI):(rDNA) (BlpI)::LEU2 leu1-32 ura4-D18 ade6-DN/N abo1Δ::kanR*
***abo2Δ* series**	
LJ105	*mat3-M (EcoRV)::ade6*$^{+}$ *(BlpI)::LEU2 abo2Δ::kanR leu1-32 ura4-D18 ade6-DN/N*
DB52	*(XmnI)::ura4*$^{+}$ *mat2-P mat3-M (EcoRV)::ade6 IR-RΔ (SpeI)::(STAR1) (BlpI)::LEU2 leu1-32 ura4-D18 ade6-DN/N abo2Δ::kanR*
DB53	*(XmnI)::ura4*$^{+}$ *mat2-P mat3-M (EcoRV)::ade6 IR-RΔ (SpeI)::(STAR2) (BlpI)::LEU2 leu1-32 ura4-D18 ade6-DN/N abo2Δ::kanR*
DB49	*(XmnI)::ura4*$^{+}$ *mat2-P mat3-M (EcoRV)::ade6 IR-RΔ (SpeI)::(STAR3) (BlpI)::LEU2 leu1-32 ura4-D18 ade6-DN/N abo2Δ::kanR*
DB51	*(XmnI)::ura4*$^{+}$ *mat2-P mat3-M (EcoRV)::ade6 IR-RΔ (SpeI)::(STAR4) (BlpI)::LEU2 leu1-32 ura4-D18 ade6-DN/N abo2Δ::kanR*
DB50	*(XmnI)::ura4*$^{+}$ *mat2-P mat3-M (EcoRV)::ade6 IR-RΔ (SpeI):(BTH1) (BlpI)::LEU2 leu1-32 ura4-D18 ade6-DN/N abo2Δ::kanR*
DB54	*(XmnI)::ura4*$^{+}$ *mat2-P mat3-M (EcoRV)::ade6 IR-RΔ (SpeI):(rDNA) (BlpI)::LEU2 leu1-32 ura4-D18 ade6-DN/N abo2Δ::kanR*
**Strains in Figure S11**	
***gcn5Δ* series**	
PA91	*(XmnI)::ura4*$^{+}$ *mat2-P mat3-M (EcoRV)::ade6*$^{+}$ *gcn5Δ::kanR leu1-32 ura4-D18 ade6-DN/N*
PA92	*(XmnI)::ura4*$^{+}$ *mat2-P mat3-M (EcoRV)::ade6*$^{+}$ *IR-RΔ (SpeI)::(STAR1) (BlpI)::LEU2 gcn5Δ::kanR leu1-32 ura4-D18 ade6-DN/N*
PA93	*(XmnI)::ura4*$^{+}$ *mat2-P mat3-M (EcoRV)::ade6*$^{+}$ *IR-RΔ (SpeI)::(STAR2) (BlpI)::LEU2 gcn5Δ::kanR leu1-32 ura4-D18 ade6-DN/N*
PA94	*(XmnI)::ura4*$^{+}$ *mat2-P mat3-M (EcoRV)::ade6*$^{+}$ *IR-RΔ (SpeI)::(STAR3) (BlpI)::LEU2 gcn5Δ::kanR leu1-32 ura4-D18 ade6-DN/N*
PA95	*(XmnI)::ura4*$^{+}$ *mat2-P mat3-M (EcoRV)::ade6*$^{+}$ *IR-RΔ (SpeI)::(STAR4) (BlpI)::LEU2 gcn5Δ::kanR leu1-32 ura4-D18 ade6-DN/N*
PA96	*(XmnI)::ura4*$^{+}$ *mat2-P mat3-M (EcoRV)::ade6*$^{+}$ *IR-RΔ (SpeI)::(BTH1) (BlpI)::LEU2 gcn5Δ::kanR leu1-32 ura4-D18 ade6-DN/N*
PA97	*(XmnI)::ura4*$^{+}$ *mat2-P mat3-M (EcoRV)::ade6*$^{+}$ *IR-RΔ (SpeI)::(rDNA) (BlpI)::LEU2 gcn5Δ::kanR leu1-32 ura4-D18 ade6-DN/N*
PA98	*(XmnI)::ura4*$^{+}$ *mat2-P mat3-M (EcoRV)::ade6*$^{+}$ *IR-RΔ (SpeI)::(edge cen1) (BlpI)::LEU2 gcn5Δ::kanR leu1-32 ura4-D18 ade6-DN/N*

not2Δ **series**	
DB105	*mat3-M (EcoRV)::ade6*$^+$ *(BlpI)::LEU2 not2Δ::kanR leu1-32 ura4-D18 or DS/E ade6-DN/N*
DB4	*(XmnI)::ura4*$^+$ *mat2-P mat3-M (EcoRV)::ade6*$^+$ *IR-RΔ (SpeI)::(STAR1) (BlpI)::LEU2 not2Δ::kanR leu1-32 ura4-D18 ade6-DN/N*
DB5	*(XmnI)::ura4*$^+$ *mat2-P mat3-M (EcoRV)::ade6*$^+$ *IR-RΔ (SpeI)::(STAR2) (BlpI)::LEU2 not2Δ::kanR leu1-32 ura4-D18 ade6-DN/N*
DB1	*(XmnI)::ura4*$^+$ *mat2-P mat3-M (EcoRV)::ade6*$^+$ *IR-RΔ (SpeI)::(STAR3) (BlpI)::LEU2 not2Δ::kanR leu1-32 ura4-D18 ade6-DN/N*
DB3	*(XmnI)::ura4*$^+$ *mat2-P mat3-M (EcoRV)::ade6*$^+$ *IR-RΔ (SpeI)::(STAR4) (BlpI)::LEU2 not2Δ::kanR leu1-32 ura4-D18 ade6-DN/N*
DB2	*(XmnI)::ura4*$^+$ *mat2-P mat3-M (EcoRV)::ade6*$^+$ *IR-RΔ (SpeI)::(BTH1) (BlpI)::LEU2 not2Δ::kanR leu1-32 ura4-D18 ade6-DN/N*
DB6	*(XmnI)::ura4*$^+$ *mat2-P mat3-M (EcoRV)::ade6*$^+$ *IR-RΔ (SpeI)::(rDNA) (BlpI)::LEU2 not2Δ::kanR leu1-32 ura4-D18 ade6-DN/N*
DB7	*(XmnI)::ura4*$^+$ *mat2-P mat3-M (EcoRV)::ade6*$^+$ *IR-RΔ (SpeI)::(edge cen1) (BlpI)::LEU2 not2Δ::kanR leu1-32 ura4-D18 ade6-DN/N*
cph1Δ **series**	
DN121	*mat3-M (EcoRV)::ade6*$^+$ *(BlpI)::LEU2 cph1Δ::kanR leu1-32 ura4-D18 or DS/E ade6-DN/N*
DN117	*(XmnI)::ura4*$^+$ *mat2-P mat3-M (EcoRV)::ade6*$^+$ *IR-RΔ (SpeI)::(STAR1) (BlpI)::LEU2 cph1Δ::kanR leu1-32 ura4-D18 or DS/E ade6-DN/N*
DN118	*(XmnI)::ura4*$^+$ *mat2-P mat3-M (EcoRV)::ade6*$^+$ *IR-RΔ (SpeI)::(STAR2) (BlpI)::LEU2 cph1Δ::kanR leu1-32 ura4-D18 or DS/E ade6-DN/N*
DN114	*(XmnI)::ura4*$^+$ *mat2-P mat3-M (EcoRV)::ade6*$^+$ *IR-RΔ (SpeI)::(STAR3) (BlpI)::LEU2 cph1Δ::kanR leu1-32 ura4-D18 or DS/E ade6-DN/N*
DN116	*(XmnI)::ura4*$^+$ *mat2-P mat3-M (EcoRV)::ade6*$^+$ *IR-RΔ (SpeI)::(STAR4) (BlpI)::LEU2 cph1Δ::kanR leu1-32 ura4-D18 or DS/E ade6-DN/N*
DN115	*(XmnI)::ura4*$^+$ *mat2-P mat3-M (EcoRV)::ade6*$^+$ *IR-RΔ (SpeI)::(BTH1) (BlpI)::LEU2 cph1Δ::kanR leu1-32 ura4-D18 or DS/E ade6-DN/N*
DN119	*(XmnI)::ura4*$^+$ *mat2-P mat3-M (EcoRV)::ade6*$^+$ *IR-RΔ (SpeI)::(rDNA) (BlpI)::LEU2 cph1Δ::kanR leu1-32 ura4-D18 or DS/E ade6-DN/N*
DN120	*(XmnI)::ura4*$^+$ *mat2-P mat3-M (EcoRV)::ade6*$^+$ *IR-RΔ (SpeI)::(edge cen1) (BlpI)::LEU2 cph1Δ::kanR leu1-32 ura4-D18 or DS/E ade6-DN/N*
rcd1Δ **series**	
DB112	*mat3-M (EcoRV)::ade6*$^+$ *(BlpI)::LEU2 rcd1Δ::kanR leu1-32 ura4-D18 or DS/E ade6-DN/N*
DB60	*(XmnI)::ura4*$^+$ *mat2-P mat3-M (EcoRV)::ade6*$^+$ *IR-RΔ (SpeI)::(STAR1) (BlpI)::LEU2 rcd1Δ::kanR leu1-32 ura4-D18 ade6-DN/N*
DB61	*(XmnI)::ura4*$^+$ *mat2-P mat3-M (EcoRV)::ade6*$^+$ *IR-RΔ (SpeI)::(STAR2) (BlpI)::LEU2 rcd1Δ::kanR leu1-32 ura4-D18 ade6-DN/N*
DB57	*(XmnI)::ura4*$^+$ *mat2-P mat3-M (EcoRV)::ade6*$^+$ *IR-RΔ (SpeI)::(STAR3) (BlpI)::LEU2 rcd1Δ::kanR leu1-32 ura4-D18 ade6-DN/N*
DB59	*(XmnI)::ura4*$^+$ *mat2-P mat3-M (EcoRV)::ade6*$^+$ *IR-RΔ (SpeI)::(STAR4) (BlpI)::LEU2 rcd1Δ::kanR leu1-32 ura4-D18 ade6-DN/N*
DB58	*(XmnI)::ura4*$^+$ *mat2-P mat3-M (EcoRV)::ade6*$^+$ *IR-RΔ (SpeI)::(BTH1) (BlpI)::LEU2 rcd1Δ::kanR leu1-32 ura4-D18 ade6-DN/N*
DB62	*(XmnI)::ura4*$^+$ *mat2-P mat3-M (EcoRV)::ade6*$^+$ *IR-RΔ (SpeI)::(rDNA) (BlpI)::LEU2 rcd1Δ::kanR leu1-32 ura4-D18 ade6-DN/N*
DB63	*(XmnI)::ura4*$^+$ *mat2-P mat3-M (EcoRV)::ade6*$^+$ *IR-RΔ (SpeI)::(cen1 NheI) (BlpI)::LEU2 rcd1Δ::kanR leu1-32 ura4-D18 ade6-DN/N*

***btf3Δ* series**	
DB16	*mat3-M (EcoRV)::ade6*$^{+}$ *(BlpI)::LEU2 btf3Δ::kanR leu1-32 ura4-D18 ade6-DN/N*
DB20	*(XmnI)::ura4*$^{+}$ *mat2-P mat3-M (EcoRV)::ade6*$^{+}$ *IR-RΔ (SpeI)::(STAR1) (BlpI)::LEU2 btf3Δ::kanR leu1-32 ura4-D18 ade6-DN/N*
DB21	*(XmnI)::ura4*$^{+}$ *mat2-P mat3-M (EcoRV)::ade6*$^{+}$ *IR-RΔ (SpeI)::(STAR2) (BlpI)::LEU2 btf3Δ::kanR leu1-32 ura4-D18 ade6-DN/N*
DB17	*(XmnI)::ura4*$^{+}$ *mat2-P mat3-M (EcoRV)::ade6*$^{+}$ *IR-RΔ (SpeI)::(STAR3) (BlpI)::LEU2 btf3Δ::kanR leu1-32 ura4-D18 ade6-DN/N*
DB19	*(XmnI)::ura4*$^{+}$ *mat2-P mat3-M (EcoRV)::ade6*$^{+}$ *IR-RΔ (SpeI)::(STAR4) (BlpI)::LEU2 btf3Δ::kanR leu1-32 ura4-D18 ade6-DN/N*
DB18	*(XmnI)::ura4*$^{+}$ *mat2-P mat3-M (EcoRV)::ade6*$^{+}$ *IR-RΔ (SpeI)::(BTH1) (BlpI)::LEU2 btf3Δ::kanR leu1-32 ura4-D18 ade6-DN/N*
DB22	*(XmnI)::ura4*$^{+}$ *mat2-P mat3-M (EcoRV)::ade6*$^{+}$ *IR-RΔ (SpeI)::(rDNA) (BlpI)::LEU2 btf3Δ::kanR leu1-32 ura4-D18 ade6-DN/N*
DB23	*(XmnI)::ura4*$^{+}$ *mat2-P mat3-M (EcoRV)::ade6*$^{+}$ *IR-RΔ (SpeI)::(cen1 NheI) (BlpI)::LEU2 btf3Δ::kanR leu1-32 ura4-D18 ade6-DN/N*
***ckb1Δ* series**	
DN97	*mat3-M (EcoRV)::ade6*$^{+}$ *(BlpI)::LEU2 ckb1Δ::kanR leu1-32 ura4-D18 or DS/E ade6-DN/N*
DN93	*(XmnI)::ura4*$^{+}$ *mat2-P mat3-M (EcoRV)::ade6*$^{+}$ *IR-RΔ (SpeI)::(STAR1) (BlpI)::LEU2 ckb1Δ::kanR ura4-D18 or DS/E ade6-DN/N*
DN94	*(XmnI)::ura4*$^{+}$ *mat2-P mat3-M (EcoRV)::ade6*$^{+}$ *IR-RΔ (SpeI)::(STAR2) (BlpI)::LEU2 ckb1Δ::kanR ura4-D18 or DS/E ade6-DN/N*
DN90	*(XmnI)::ura4*$^{+}$ *mat2-P mat3-M (EcoRV)::ade6*$^{+}$ *IR-RΔ (SpeI)::(STAR3) (BlpI)::LEU2 ckb1Δ::kanR ura4-D18 or DS/E ade6-DN/N*
DN92	*(XmnI)::ura4*$^{+}$ *mat2-P mat3-M (EcoRV)::ade6*$^{+}$ *IR-RΔ (SpeI)::(STAR4) (BlpI)::LEU2 ckb1Δ::kanR ura4-D18 or DS/E ade6-DN/N*
DN91	*(XmnI)::ura4*$^{+}$ *mat2-P mat3-M (EcoRV)::ade6*$^{+}$ *IR-RΔ (SpeI)::(BTH1) (BlpI)::LEU2 ckb1Δ::kanR ura4-D18 or DS/E ade6-DN/N*
DN95	*(XmnI)::ura4*$^{+}$ *mat2-P mat3-M (EcoRV)::ade6*$^{+}$ *IR-RΔ (SpeI)::(rDNA) (BlpI)::LEU2 ckb1Δ::kanR ura4-D18 or DS/E ade6-DN/N*

Table S4. Oligonucleotide sequences.

Oligonucleotide	Sequence
**Confirmation of Bioneer ORF deletions**	
Cp-N10 (GTO-596)	GATGTGAGAACTGTATCCTAGCAAG
Cp-C3 (GTO-597)	GGCTGGCCTGTTGAACAAGTCTGGA
Cp5-mcl1D	CGAGAATCTCACACAGTTCTACAGC
Cp3-mcl1D	GCCAGAACTTTGGGCGTTAGCTC
Cp5-wpl1D	GGTGGCTCAATAATGGCCGCATTCG
Cp3-wpl1D	GGCGATAACACAACCCTTTCCATC
Cp5-dpb4D	GTTCGAGGAGATGGTTAGAAGCAAGTG
Cp3-dpb4D	CTGGACAGTAAACACCGATTAGGATG
GTO-598 pli1 (03-81) c	GCTGCCCGTAGTCTAATCAATCATT
GTO-599 ckb1 (04-23) c	CTACAAAGTGTCACTGTCACACCTG
GTO-600 abo1 (05-33)	TACACGCGCATATCCTGTGTGCGTG
GTO-601 pdr13 (06-02)	GCATGTGGTGATAACACAGATAGTAG
GTO-602 mrc1 (06-19) c	GGCAACGTTTATGGTGATATGACTG
GTO-603 rhp18 (07-46)	GGTACATCTAGAAATCTTCACATGC
GTO-604 ppo1 (07-80) c	GCATTGCCAGATTGATGTAGCTTGC
GTO-605 mit1 (09-32) c	GTATCCGCAAATACCTATATGGCTC
GTO-606 ddb1 (10-52) c	GAATATCCTGTTAATGCAATCAC
GTO-607 pof3 (11-94) c	GCTTCAGTGCAAAATAGTAAACACC
GTO-608 ccq1 (14-25) c	CCTTATAAGAGCCAGAATGAATTGC
GTO-609 cdt2 (14-69) c	GGGTCCTGCAAGGTGAACAGCTAAC
GTO-610 rtf1 (14-80) c	GATATGAGGCAGATACCTGTAAACG
GTO-611 rnc1 (17-85) c	CTTCCAAAGATATAACGACTGAGGC
GTO-612 csn1 (22-54) c	GAATACATGGGGTTCTAATCTTGG
GTO-613 meu6 (26-47) c	CGCAGACAGCTCGATTCCAGAAG
GTO-614 pob3 (26-94) c	CGTAGGAGACAATATGGGAATCCCG
GTO-615 did2 (29-18) c	GGTTGAAGAGCGGTGTCCTTTGC
GTO-616 spc19 (29-55)	CCGAAGGATTGTGTAGTTTACACAGG
GTO-617 cph1 (29-75) c	GCGTTTTAAACCATTGATATGGCTG
GTO-618 fft3 (30-11) c	CTAGGTGAAGTGCTTTCCTCTGTC
GTO-619 fkbp39 (30-46) c	CTGCAATCCCTACTGGGCGTCGACGC
GTO-620 msh6 (31-89) c	CAGAGGATTGTTGGCAGGATCTTGG
GTO-621 wpl1 (32-77) c	GCTGTAAACGAGTACATAGCCATGG
**PCR amplification of *mcl1Δ::kanR*, *wpl1Δ::kanR* and *dpb4Δ::kanR***	
GTO-1332	CCTGTGAGGCTCCACTAGGTTTCTC
GTO-1333	CTGTTGCTCGTTCAATTCATCGCCTAC
GTO-1363	CTAGTTATAAAGGGAAGTCGGTCTTCAC
GTO-1364	CTTCTGCTTTCACTGGAATAGCAACATC
GTO-1365	GAGCGTGTCGCGTTTGTTGACGCGTCGC
GTO-1366	CGTTATGGCGAGCTGAAGCTTCTGTCC
**RT-QPCR for *ade6* and *act1***	
GTO-218	GGCTCGAGCCCGGGCATGGAAATTGCAG
GTO-219	GGGGATCCATGGCAAAGGGTTTGAGCACG
TJO-55	CTGTTTTGTCTTTGTATGCC
TJO-58	TAAGGTAGTCAGTCAAGTCA

**Identification of *ade6-DN/N* allele**	
GTO-232	CATTGGCTTACGACGGTCGTGG
GTO-233	CCACATATGGCCCGTAAGTGAGC
